# Understanding, comprehensibility and acceptance of an evidence-based consumer information brochure on fall prevention in old age: a focus group study

**DOI:** 10.1186/1471-2318-11-26

**Published:** 2011-05-20

**Authors:** Sabine Lins, Andrea Icks, Gabriele Meyer

**Affiliations:** 1 Faculty of Health, School of Nursing Science, University of Witten/Herdecke, Stockumer Straße 12, D-58453 Witten, Germany; 2 Faculty of Medicine, Department of Public Health, Heinrich-Heine-University Düsseldorf, Moorenstraße 5, 40225 Düsseldorf, Germany

## Abstract

**Background:**

Evidence-based patient and consumer information (EBPI) is an indispensable component of the patients' decision making process in health care. Prevention of accidental falls in the elderly has gained a lot of public interest during preceding years. Several consumer information brochures on fall prevention have been published; however, none fulfilled the criteria of an EBPI. Little is known about the reception of EBPI by seniors. Therefore we aimed to evaluate a recently developed EBPI brochure on fall prevention with regard to seniors' acceptance and comprehensibility in focus groups and to explore whether the participants' judgements differed depending on the educational background of the study participants.

**Methods:**

Seven focus groups were conducted with 40 seniors, aged 60 years or older living independently in a community. Participants were recruited by two gatekeepers. A discussion guide was used and seniors were asked to judge the EBPI brochure on fall prevention using a Likert scale 1-6. The focus group discussions were tape recorded, transcribed verbatim, and analysed using content analysis.

**Results:**

The participants generally accepted the EBPI brochure on fall prevention. Several participants expressed a need for more practical advice. The comprehensibility of the brochure was influenced positively by brief chapter summaries. Participants dismissed the statistical illustrations such as confidence intervals or a Fagan nomogram and only half of them agreed with the meta-information presented in the first chapter. The detailed information about fall prevalence was criticised by some seniors. The use of a case story was well tolerated by the majority of participants.

**Conclusion:**

Our findings indicate that the recently developed EBPI brochure on fall prevention in old age was generally well accepted by seniors, but some statistical descriptions were difficult for them to understand. The brochure has to be updated. However, not all issues raised by the participants will be taken into account since some of them are contrary to the principles of EBPI.

## Background

Ethical guidelines demand that evidence-based, clear and unbiased information is offered and made available to all patients [1]. Individuals' needs should be targeted and best available evidence should be provided using principles of risk communication and plain language [2].

Surveys have demonstrated that patients have preferences towards involvement in informed decision making [3]. However, studies suggest that information which has been gathered from patient information brochures or the world-wide-web or which has been provided orally by health carers is not evidence-based [4,5]. Analyses have shown that conventional information often neglects presentation of the lack of benefit and adverse effects of the interventions discussed [4-6] and messages are offered in a persuasive or oversimplified manner [7,8].

It has been proven that evidence-based patient information (EBPI) effectively empowers patients' informed decision making in acute and chronic conditions [9-11]. EBPI aims to present current best evidence on benefit and harm of treatment and diagnostic options using the principles of risk communication and plain language [2]. The latter implies use of everyday language, active voice and short sentences, illustrated by bullets and tables [12].

Empirical evidence on the acceptability of EBPI dealing with preventive options by patients is sparse. Reports on negative response towards EBPI are even more sparse [13]. It has been suggested that cognitive dissonance could play a role, indicating that patients choose information according to their attitudes and react with strong rejection of opposed information [13].

During preceding years prevention of accidental falls in the elderly has been given much attention in research and practice. National preventive programmes have been implemented [14] and information brochures on fall risk and fall prevention have been published [15-17] but none of the brochures fulfil the criteria of EBPI (Table 1) [2]. Therefore we recently developed an EBPI on risk and prevention of accidental falls. The aim of this study was to evaluate its comprehensibility and acceptability in focus groups with healthy senior volunteers and to explore whether the judgement differed depending on the educational background of the study participants.

**Table 1:** Pool of EBPI categories [2].

**EBPI categories with short explanations**	
-	Content of information and meta-information
	*Description of how patients should be informed before medical interventions and which criteria of transparency should be considered.*
-	Quality of evidence
	*Authors should use a clear system for grading the quality of evidence and for the representation of strength of recommendation.*
-	Patient-oriented outcome measures
	*Patient-oriented or disease-oriented outcomes should be included.*
-	Presentation of numerical data
	*Existing evidence about the way how numerical data is presented should be considered.*
-	Verbal presentation of risk
	*Risk communication should comprise not only numerical but also verbal descriptors.*
-	Diagrams, graphics and charts
	*An adequate way of representing numerical information should be chosen.*
-	Loss and gain framing
	*Information on gain and loss should be represented in a balanced manner.*
-	Pictures and drawings
	*Written text should be combined with appropriate cartoons, pictures, pictograms, drawings, and photographs.*
-	Patient narratives
	*Patient narratives are assumed to improve comprehensibility and memorability of information.*
-	Consideration of cultural aspects
	*Health information should consider cultural aspects such as religiousness, masculinity versus femininity.*
-	Layout
	*Existing evidence about how the layout can support reading and comprehension should be considered.*
-	Language
	*Plain language in a non-alarmist and non-patronising way is recommended for enhancing understanding.*
-	Development process
	*Consumers should be involved in the development process of the information.*

## Methods

### Development and description of the brochure

Our brochure does not comprise recommendations on how to prevent accidental falls, but aims to present current scientific evidence on risk of falling and benefit and lack of benefit of different preventive approaches, in a comprehensive and non-persuasive manner. In 2007, a first version of an EBPI brochure was developed and evaluated in focus groups with 19 seniors [18]. The evaluation revealed the brochure's limited acceptance due to unfamiliarity with graphical figures and difficulties in understanding of risk communication. Subsequently, the brochure underwent intensive modification. Evidence was updated, tables and figures were revised, a case story was included, and a guideline for plain language was taken into account [12]. The second version of the brochure comprises 57 pages, eight chapters and eight tables and figures, including a bar chart, a pictogram, and a Fagan nomogram. The brochure comprises information on the definition of falls, fall risk factors, gender and age related differences and fall related consequences (Table 2). The brochure is available as additional file 1 to this article. It is currently being updated.

**Table 2:** Chapters and Content of the Evidence-Based Patient Information Brochure on Risk of Accidental Falls.

**Chapter (length)**	**Content**	**Presentation**	**Fulfilment of EBPI categories **[2]
One (6 pages)	Introduction	Narrative	Meta-information (e.g.: information about the authors, sponsoring or financial support, global aim of the publication, sources of information used, publication date), development process
Two (6 pages)	Definition of falls, fall prevalence in different groups (age, gender)	Narrative, bar chart, tables, line graphs, summary	Presentation of numerical data, diagrams, graphics and charts, patient narratives, layout, language
Three (13 pages)	Identifying the individual risk of falling	Narrative, case story, individual risk of falling table with confidence intervals, nomogram, summary	Presentation of numerical data, verbal presentation of risk, diagrams, graphics and charts, patient narratives, layout, language
Four (17 pages)	Fall preventive interventions	Narrative, case story, drawings, pictograph, table displaying the number need to treat (NNT), summary	Content of information, quality of evidence, patient-oriented outcome measures, presentation of numerical data, verbal presentation of risk, diagrams, graphics and charts, loss and gain framing, pictures and drawings, patient narratives, layout, language
Five (1 page)	Final remarks, offering the opportunity to give feedback, referral to the following chapters	Narrative	Not addressed by EBPI categories
Six (3 pages)	Further literature and contact persons	Narrative	Meta-information
Seven (5 pages)	Glossary	Narrative	Language
Eight (3 pages)	References	Narrative	Meta-information

### Design

In order to explore participants' individual perceptions of the brochure, we chose the method of focus group discussion. Since we evaluated participants' opinions evolved by the social interaction in the focus group, our study could be assigned to the theoretical perspective of symbolic interactionism. Focus groups are recommended for the evaluation of material for risk clarification [19]. The focus group method has several advantages. This method is useful for examining what participants think and why they think this way. It is possible to generate aspects which are important for the reader, in their own words and with their own ranking [20]. The researcher can check his interpretation by asking the participants during the discussion. Another benefit of the focus group is the concurrent elicitation of a number of opinions [21].

### Sampling and Recruitment

In 2008, two managers from two community centres for elderly people in Freiburg im Breisgau, Germany, were asked to act as gatekeepers for purposeful sampling. They selected eligible seniors who complied with predefined inclusion criteria: age ≥ 60 years and living independently in a community. The gatekeepers were asked to explicitly consider the different educational backgrounds of seniors in order to obtain maximum variation sampling. The investigator regularly reviewed the demographic data of participants throughout the consecutive recruitment process in order to ensure inclusion of different educational backgrounds. Exclusion criteria were: living in a nursing home or receiving formal professional nursing care at home. The gatekeepers contacted existing groups of seniors and asked if the study could be introduced to them. The investigator visited the community centres to explain the purpose of the study to those who were interested in participating. The brochure was handed out to all eligible participants at least one week before the focus group took place. Careful reading was requested. In total, 40 participants (31 women and 9 men) agreed to participate. Seven focus groups were conducted with three to ten participants.

### Setting and data collection

The focus groups were conducted by one investigator (SL), lasted 90 to 120 minutes, and took place in quiet rooms at the two community centres. To provide a pleasant atmosphere, refreshments and sweets were offered. Data collection was performed between April 2009 and June 2009. The main purpose of the focus groups was to explore participants' understanding and acceptability of the brochure. A pre-tested focus group discussion guide was used [18] and the focus groups were audio-taped. For warming up, participants were asked to talk about their own experience with falling or with fall risk. Each group session was then opened with a short introduction, asking the participants to express their first impression of the brochure. Each chapter, the tables and figures, and the overall impression of the brochure were discussed. Open questions were used. Thus participants had the opportunity to highlight aspects which seemed to be most important to them. The investigator encouraged participants to make suggestions on how to improve the content of the brochure. Participants' answers with a nod were verbalized to make it audible for the audio recording. After discussion of each chapter, participants were asked for an overall judgement of the chapter using a Likert scale ranging from "1 = very good" to "6 = insufficient". At the end of the focus group, participants completed a single page questionnaire on socio-demographic characteristics.

### Data analysis and ethical considerations

The audiotapes were transcribed verbatim but anecdotes, jokes and teasing were not taken into account. Only comments relevant to the research question were transcribed. The transcripts were analysed using content analysis. Names of participants were replaced with codes to assure anonymity. It was noted if a participant agreed to the comment of another participant. A second experienced researcher not involved in the study checked all transcripts for accuracy of comments, right quantity of approbations and first categorisation.

The transcript analysis covered three steps: First, the focus group discussions were separated into sections according to the chapter discussed. Within these sections, the researcher categorised each statement as "positive", "negative", "neutral" or "suggestion".

This assignment was useful for the following analytic process because no intonation or speaking pause were transcribed. Consecutively, qualitative data analysis software [22] was used. The second step involved open coding [23]. The open coding was influenced by the themes of the topic guide and the themes which were addressed by the participants during the discussion. Within the codes the classification of "positive", "negative", "neutral" and "suggestion" remained as sub-codes. In a third step the meaning of the participants' statements were summarised at the sub-codes. This required an intensive familiarisation with the data. If possible, the participants' own words were used in the summary. Alternative comprehensive terms were applied. Statements mentioned in more than one focus group were given more weight than statements which emerged in only one focus group. The participants' suggestions were added to the different chapters. Likert scale ratings and socio-demographic data were analysed through descriptive statistics using 'SPSS^® ^Statistics 17.0' and 'Microsoft Excel' by Microsoft Windows. Whether participants' judgement differed depending on the educational background was examined by grouping participants into five categories according to their professional education (Table 3). The mean Likert scale rating of each group for each chapter was calculated. Results were illustrated with Excel and compared. The protocol was approved by the ethics committee of the German Society of Nursing Science. Participants were orally informed about the study, received written information and were asked to sign a written informed consent sheet.

**Table 3:** Participants' characteristics (n = 40).

	**n (%)**
Women	31 (78)
Mean age (range), yrs.	75 (60-89)
Living alone	22 (55)
Education	
none	1 (2.5)
secondary modern school (graduation after 9 school years, lower than a high school diploma)	16 (40)
high-school diploma	12 (30)
college qualification	1 (2.5)
general qualification for university entrance	8 (20)
other	2 (5)
Professional education	
none	2 (5)
semiskilled	5 (12.5)
vocational training	27 (67.5)
university	6 (15)
Current employment status and source of income	
≥ part-time position	1 (2.5)
yes, additional income to the old age pension	1 (2.5)
no, housewife/house husband	7 (17.5)
no, pension	30 (75)
no, other	1 (2.5)
Occupational*	
Blue-collar employee	4 (10)
(weekly contract and temporary workers)	
White-collar employee	25 (62.5)
(permanent salaried employees)	
civil servant	6 (15)
self-employed	3 (7.5)
other	1 (2.5)
Current net income in EUR^†^	
≥ 1000	13 (32.5)
1000 € to 2000	11 (27.5)
2000 € to 3000	8 (20)
3000 € to 4000	4 (10)

* One missing value.

^† ^Four missing values.

## Results

### Population

Participants' characteristics are displayed in Table 3. Mean age was 75 years, 78% were female. The majority of participants had graduated with a high-school diploma (30%) or with the lower levelled secondary modern school qualification after 9 years schooling (40%). Two thirds of the participants had passed vocational training (67.5%).

### Themes

Four themes emerged during focus group discussion and 21 were pre-determined by the elements of the brochure (Table 4). In the following section we focus on themes of high relevance for the development of patient information according to internationally discussed EBPI requirements. The other themes will not be considered since they deal with specific fall prevention issues and understanding of scientific language in German only. Each comment is presented alongside with the number of participants who raised this issue. So the reader will get an impression as to whether it is the opinion of a single person or of a number of seniors from different focus groups.

**Table 4:** Codes defined by elements of Evidence-Based Patient Information Brochure on Risk of Accidental Falls and codes identified through focus group discussions.

Codes generated from pre-defined topics of the brochure (sub-codes)	Codes emerged during focus group discussions
Cover picture, layout, chapter 1 (including sub-code: meta-information and development process), table one, figure one, chapter 2 (including the sub-code: fall prevalence), table two, figure two, chapter 3 (including the sub-code: case story), table three, nomogram, chapter 4, table four, pictogram, further literature and contact persons, glossary, overall acceptance (including the sub-code: practical advice), expectations to the brochure, recommendation of the brochure, emotional reactions	Marginal notes, routes of dissemination; facilitators target group, usage of 1000 persons as denominator

### Overall acceptance

In general, participants appreciated the EBPI brochure on fall prevention. They assessed the value of the brochure as "very good" (7 participants/4 focus groups) and "good" (12/3). One participant of the focus group (FG) 7 stated: "A very informative and recommendable aid for seniors and affected persons." This judgement is also reflected by the Likert scale ratings (Table 5). Two participants had a negative opinion towards the brochure. One participant judged the brochure as boring and another called the brochure worthless. Participants approved the length of the brochure (6/5) and emphasised their increased knowledge after reading the whole brochure (11/4) as well as the summaries of the chapters (13/4). However, a number of participants criticised the brochure for providing too many statistics (16/4) and not enough practical hints (22/4). No one raised any strong rejection of the brochure.

**Table 5:** Likert scale rating of the brochure's chapters.

		**Focus group (number of participants)**						
**Chapter**	**Mean value of chapter judgement, calculated**	**1 (n = 10)**	**2 (n = 7)**	**3 (n = 6)**	**4 (n = 6)**	**5 (n = 4)**	**6 (n = 3)**	**7 (n = 4)**
**1**	2.68	4.04	2.64	2.20	1.17	2.88	2.00	3.83
**2**	2.75	3.55	2.07	2.33	2.00	2.00	3.33	4.00
**3**	2.87	3.90	3.36	2.00	1.75	2.25	3.00	3.83
**4**	2.48	2.70	2.86	1.75	1.83	2.25	2.00	4.00
**6 & 7**	2.29	2.10	2.36	2.00	2.08	2.00	2.00	3.50
**Overall judgement (inquired)**	2.43	Missing value*	3.00	2.00	2.00	1.75	2.00	3.83

1 = very good, 2 = good, 3 = satisfactory, 4 = adequate, 5 = inadequate, 6 = insufficient.

* Not available since the investigator failed to ask participants.

### Meta-information and development process

Meta-information and a short description of the development process of the brochure have been presented in chapter one. Meta-information comprises criteria for supporting the transparency of the brochure's development such as authors' names and affiliation, sponsoring, financial support, global aim of the publication, sources of information used, and publication date [2]. Some participants agreed to the meta-information (13/2). One participant of FG 3 commented: "Introduction is good, since it comprises suggestions about what we should pay attention to." However, other participants did not agree with the information (11/2). The first chapter was more often judged as redundant (7/3) than useful (2/2). No clear trend could be documented concerning a level of detail offered in the first chapter.

### Fall prevalence

Fall prevalence of different risk groups related to age and gender was presented and displayed in bar charts, tables, and line graphs. Some participants judged the differentiation of fall, fall-related injury, and gender related risk of hip fracture as far too detailed (2/2). One senior from FG 5 stated his opinion as follows: "Why in the world do you list in such great detail who falls and why they all fall. That's not exactly inviting. Is that necessary? Is that part of the scientific procedure?" Repetition and double presentation as narrative text alongside graphic displays were judged negatively (4/2). The single item of information on 30% to 40% of elderly persons falling at least once a year alongside a single statement on the percentage of falls in younger age groups was considered to be sufficient (1/1). Most participants understood the bar chart (28/5) and the line graph (9/4). Tables displayed in the brochure caused more difficulties. A table displaying reasons for accidental injuries (11/2) and another table on age and gender related differences in the number of injuries due to falls caused problems (10/3).

### Case story

A case story about an elderly community dwelling woman illustrates the steps of determining the personal fall risk using a Fagan nomogram as well as decision making on fall preventive options according to personal preferences.

The majority of comments regarding the significance of the case story were positive (10/4). One participant of FG 5 explained: "I like the example for discerning the risk of falling because the reader is being introduced to the topic." A number of participants suggested presenting more than one case study (10/1).

### Using 1000 persons as denominators

Some participants had problems understanding absolute risk communication using groups of 1000 persons and would have preferred reference groups of 100 persons (4/1). One senior from FG 4 expressed his problems: "Why always refer to 1000 people? I find that so hard to visualise. If you say 'of 100', that gives me an idea."

### Calculation of individual fall risk using a table and Fagan nomogram

**Figure 1:**
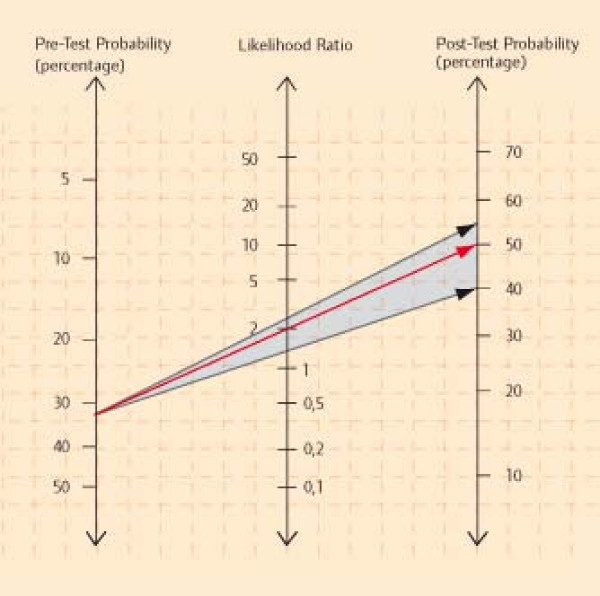
**Pre-test-post-test probability of falling using a Fagan nomogram**. Red arrow = LR+ 2; black arrows = 95% confidence interval 1.5-2.7. Original in German [32]

Participants were able to calculate their individual fall risk using a table and a nomogram. One table displayed the likelihood of falling during the next 12 months. An individual's risk of falling, assuming a 30% baseline risk, was displayed using likelihood ratios (LR) and 95% confidence intervals of empirically proven risk factors [24]. A nomogram exemplarily visualised the pre-test-post-test probability of falling, using LR+ of 2 (95% confidence interval 1.5 to 2.7). Some participants disagreed with the table because of its complexity (5/2). One senior from FG 7 stated his problems: "As an older person, table three completely overtaxes you." Few participants liked it (3/3). Only one person stated that he understood the margin of uncertainty (1/1). Participants mentioned their problems with understanding the whole table (2/2), the confidence interval (1/1) and the many numerals (1/1). The nomogram (Figure 1) aimed at illustrating the confidence interval; however, most of the participants who gave a comment did not understand it (15/4) and judged it to be redundant (8/4). One participant from FG 2 revealed: "Didn't get the figure even after reading it twice." Only three participants were able to explain it.

### Practical advice

The introduction pointed out that the brochure was not a practical guide. Nevertheless, a number of participants explicitly expressed their wish to receive more practical advice on removal of carpets, home modification like installing grab handles in the bathrooms, eliminating thresholds, not waxing the parquet floor, using a ladder instead of an unsteady chair for hanging curtains and wearing sturdy shoes (13/2), and one participant from FG 2 criticised: "Too little reasonable advice." One person asked for the description of specific exercises (1/1).

### Pictogram

The pictogram displaying 1000 stick-figures divided into three groups illustrated the effectiveness of counselling on home environment modification to prevent falls: Over 12 months 414 out of 1000 seniors experienced at least one accidental fall despite counselling, 372 of these 1000 seniors would not have fallen anyway, and 214 seniors had a benefit because they did not fall due to the counselling. The majority of the participants who gave a comment judged the pictogram as redundant (9/4), confusing and even not suitable to the needs of elderly people (8/1). Two seniors stated: "Figure four (pictogram) makes a reader feel treated as if he is in first or second grade." (FG 1); "I racked my brain over page 38 (pictogram). Why are there all those people?" (FG 4). Other participants liked the pictogram (10/2). Some participants argued that it is obvious that interventions can never be a hundred percent prevention of a fall (4/2). Participants suggested a table presenting numbers instead of the pictogram (2/2).

### Further information and glossary

Participants agreed to the information about further reading, addresses and the glossary. They appreciated the length of both chapters (9/3) and judged them as interesting and informative (3/2). Two seniors from FG 7 commented: "Chapter 6 (further information) I also find interesting as a piece of information."; "Chapter 7 (glossary) is quite interesting for one's information." Some participants claimed not to have used these chapters (11/3). Two participants found both chapters easy to understand (2/1).

### Rating within groups of different educational levels

In order to explore the different judgements in relation to participants' educational background, the sample was divided into five groups: no job training, semiskilled, apprenticeship, training college and university. The judgements of the educational groups were set in relation to the average Likert scale rating assigned. It turned out that the different educational groups rated the brochure almost identically. Participants with a university degree or apprenticeship and semiskilled persons rated the brochure with a mean Likert scale rating of 2.5. Participants who visited a training college and participants without job training assigned a mean Likert scale rating of 3.

## Discussion

Most of the participants rated the EBPI brochure positively in their overall judgement and on the Likert scale. Thus the EBPI brochure on fall prevention was evaluated as acceptable by healthy seniors living in community dwellings. Acceptance of participants with different educational levels did not differ largely. The concept of likelihood of falling, 95% confidence intervals and the corresponding nomogram caused difficulties in understanding. The majority of participants preferred less statistics.

Our findings are not surprising since Gigerenzer and colleagues verified a common statistical illiteracy for patients [25]. The question arises whether the confidence interval and the nomogram have been described sufficiently clear, or whether EBPI should be offered differently and statistically elaborated, enabling patients to choose the preferred version. EBPI should consider the special needs of the target audience [2]. Maybe the needs of the target group of the brochure were too heterogeneous to address all of them using only one brochure.

Although meta-information comprising the aim of the study and criteria of transparency has been demanded as a prerequisite of EBPI [2,26], only half of the focus group participants who commented on this theme acknowledged the information. Alternatively, meta-information could be presented in a concise table. Thus the introduction would be less extensive but the information would still be available.

It was not verified whether the insertion of a case story increases the comprehensibility of EBPI [27,28]. Most of the focus group participants appreciated the case story.

The pictogram was not judged as being helpful, a result which was confirmed by an earlier focus group evaluation [29]. However, other results demonstrated that visual data about percentage are well received and understood [30]. Other studies suggest that age might have an influence on acceptance and understanding of risk information. Further research is needed [31].

The second, explorative aim of the study was to evaluate whether study participants' judgement differed in relation to their educational background. The results suggest a slight difference between the Likert scale ratings of the group holding university degrees and the group without any professional training. This small tendency does not provide sufficient evidence to conclude that participants with a higher education rate the brochure higher. Thus it remains more or less unclear whether the educational background influences participants' judgement and acceptance of the brochure.

However, the study has several limitations. Our research is qualitative, thus the results must not be interpreted as a generalisation of the overall preferences of seniors towards EBPI. The confirmability of study results is slightly limited. A triangulation was not used to reduce effects of a potential investigator bias. The investigator could not document which statement had been raised by which participant. Therefore it was impossible to match statements and opinions to socio-demographic details. Only Likert scale results could be matched to particular participants.

The credibility of the study is good because of the relatively large sample of participants as well as a skilled investigator with sound experience in focus group methods, and support by a second investigator who analysed the transcripts for accuracy of comments and the first categorisation. Nevertheless, participants were recruited by gatekeepers and not by random sampling. Thus a sampling bias with participants having positive preferences, attitudes and opinions towards fall prevention cannot be excluded. Only a few seniors with academic education participated in our focus groups. Therefore the influence of the educational background could not be sufficiently explored.

For warming up, participants were asked to talk about their own experiences with falling or risk of falling. These data were not analysed but they could have provided interesting information about seniors' general attitudes towards falling.

The strength of the study is its transferability since detailed information is presented about the context of data collection and participants' characteristics. We tried to address the dependability by providing an extensive description of data collection and analysis.

## Conclusion

In conclusion, the majority of participating seniors acknowledged the EBPI brochure on fall prevention in old age. Our brochure is a valuable alternative to brochures with a persuasive and oversimplified style. The seniors liked the case story, disliked the nomogram visualisation, had difficulties in understanding of confidence intervals and requested the use of less statistics. The study revealed further research topics on the best way of presenting statistics to seniors, including case stories and developing EBPI using different statistical elaboration.

## Competing interests

The authors declare that they have no competing interests.

## Authors' contributions

AI and GM initiated the study. SL, AI and GM developed the study protocol. SL recruited and performed the focus groups and transcribed the audio recordings. SL interpreted the data, supported by GM and AI. SL wrote the paper with contribution of GM. AI and GM commented on paper drafts in all stages. All authors read and approved the final manuscript. SL and GM are guarantors for the paper.

## Pre-publication history

The pre-publication history for this paper can be accessed here:

http://www.biomedcentral.com/1471-2318/11/26/prepub

## Supplementary Material

Additional file 1 **Stürze und ihre Folgen: Risiko erkennen und vermeiden. Eine wissensbasierte Information für ältere Menschen [Falls and their consequences: **Realizing and preventing the risk of falling. An evidence based information for the elderly.]. Ärztekammer Nordrhein, Universität Witten/Herdecke; 2009. Evidence-based patient information brochure on risk of accidental falls investigated in the focus group discussions. Click here for file
