# Supplementary material for: Understanding, comprehensibility and acceptance of an evidence-based consumer information brochure on fall prevention in old age: a focus group study
Source: BMC Geriatr. 2011 May 20;11:26. doi: 10.1186/1471-2318-11-26 (PMC3118104; doi:10.1186/1471-2318-11-26)
Supplement: Additional file 1 — Stürze und ihre Folgen: Risiko erkennen und vermeiden. Eine wissensbasierte Information für ältere Menschen [Falls and their consequences: Realizing and preventing the risk of falling. An evidence based information for the elderly.]. Ärztekammer Nordrhein, Universität Witten/Herdecke; 2009. Evidence-based patient information brochure on risk of accidental falls investigated in the focus group discussions. [file 1471-2318-11-26-S1.PDF]

Ärztekammer Nordrhein
Körperschaft des öffentlichen Rechts

universität Witten/Herdecke

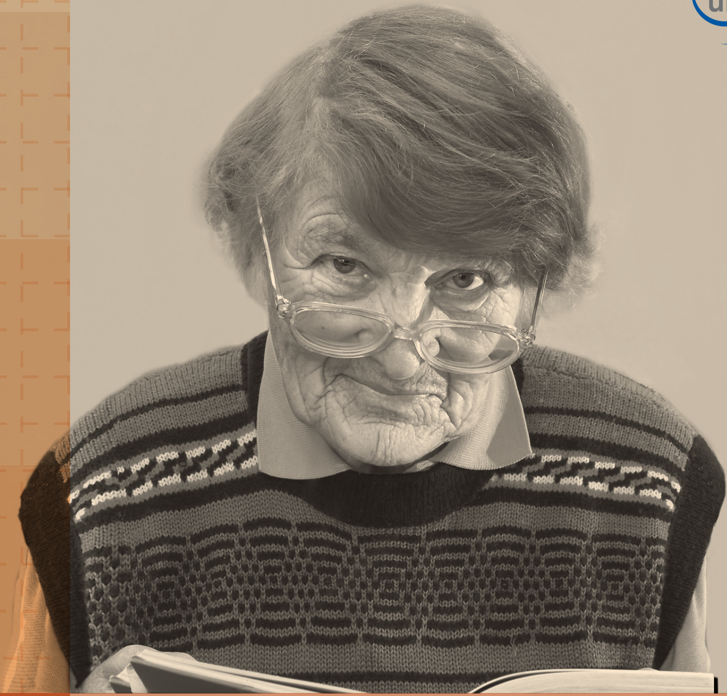

Stürze und ihre Folgen: Risiko erkennen und vermeiden

Eine wissensbasierte Information für ältere Menschen

Ärztekammer Nordrhein
Körperschaft des öffentlichen Rechts

universität Witten/Herdecke

Stürze und ihre Folgen: Risiko erkennen und vermeiden

Eine wissensbasierte Information für ältere Menschen

Impressum

Redaktion:

Ärztekammer Nordrhein, Stabstelle Kommunikation

Universität Witten/Herdecke, Fakultät für Medizin, Institut für Pflegewissenschaft

Autorinnen:

Andrea Icks, Priv.-Doz. Dr. med. Dr. P.H., Ärztin und Gesundheitswissenschaftlerin, Gesundheitsberatung und Prävention, Ärztekammer Nordrhein

Gabriele Meyer, Univ.-Prof. Dr. phil., Professur für Klinische Pflegeforschung, Universität Witten/Herdecke

Sabine Rotzoll, BScN, Universität Witten/Herdecke

Jutta Genz, Dipl. oec. troph (FH), M.A., Ärztekammer Nordrhein

Wissenschaftliche Beratung:

Gesundheitsberatungsausschuss der Ärztekammer Nordrhein

Gestaltung:

media services

Herausgeber:

Ärztekammer Nordrhein, Universität Witten/Herdecke. 2. Auflage, 2009

Bezugsadresse:

Ärztekammer Nordrhein, Tersteegenstraße 9, 40474 Düsseldorf
Priv.-Doz. Dr. med. Dr. P.H. Andrea Icks, E-Mail: andrea.icks@aekno.de
Im Internet als pdf Download unter: http://www.aekno.de/downloads/aekno/broschuere-senioren-patienten.pdf

Übersicht

		Seite
**1.**	**An die Leserinnen und Leser**	7
**2.**	**Stürze und ihre Folgen**	13
2.1	Was ist ein Sturz?	13
2.2	Wie häufig sind Stürze?	13
2.3	Welche Folgen können Stürze haben?	14
2.4	Wie häufig sind Oberschenkelhalsbrüche?	16
2.5	Welche Folgen haben Oberschenkelhalsbrüche?	18
**3.**	**Erkennen des Sturzrisikos**	19
3.1.	Wie kann das individuelle Sturzrisiko bestimmt werden?	19
3.2	Wie hoch ist das Risiko zu stürzen?	20
3.3	Welche Risikofaktoren und Risikoindikatoren gibt es?	21
3.4	Wie hoch ist Ihr persönliches Risiko zu stürzen?	23
3.5	Welche Merkmale sind mit einem erhöhten Risiko für Oberschenkelhalsbrüche verbunden?	29
**4.**	**Maßnahmen zur Vermeidung von Stürzen und ihrer Folgen**	32
4.1	Welche Maßnahmen zur Vermeidung von Stürzen gibt es?	32
4.2	Welchen Nutzen haben Maßnahmen zur Vermeidung von Stürzen?	35
4.3	Für welche Maßnahmen fehlt ein Nachweis der Sturz-vorbeugenden Wirkung?	42

Seite

4.4 Welchen Nutzen haben Maßnahmen zur Vermeidung von Sturz-bedingten Verletzungen? 44

4.5 Eigene Vorlieben berücksichtigen? 45

4.6 Welche Nebenwirkungen haben Sturz-vorbeugende Maßnahmen? 46

5. Zum Abschluss 49

6. Weiterführende Literatur und Adressen 50

6.1 Informationen und Broschüren für Verbraucher 50

6.2 Ansprechpartner 52

7. Erklärung verwendeter Fachbegriffe 53

8. Verwendete Literatur 58

Übersicht

Abbildungs- und Tabellenverzeichnis

Seite

Abbildung 1: Personen mit Sturz in den vergangenen 2 Jahren. 14

Tabelle 1: Behandlungsbedürftige Unfallverletzungen zu Hause und in der Freizeit. 14

Tabelle 2: Anzahl von Sturz-bedingten Verletzungen bei Männern und Frauen. 16

Abbildung 2: Häufigkeit von Oberschenkelhalsbrüchen bei über 65-Jährigen. 17

Tabelle 3: Risikofaktoren und ihre Auftrittswahrscheinlichkeit für mindestens 1 Sturz in 12 Monaten. 24

Abbildung 3: Skala zum Ablesen des Risikos für einen Sturz innerhalb des nächsten Jahres. 28

Abbildung 4: Nutzen von Wohnraumanpassung. 38

Tabelle 4: Maßnahmen mit belegtem Nutzen zur Vermeidung von Stürzen. 41

1. An die Leserin und den Leser

Liebe Leserin, lieber Leser,
vielleicht ist in Ihrem Bekanntenkreis in der letzten Zeit jemand gestürzt. Vielleicht hat sich die Person sogar die Hand verstaucht oder gar den Oberschenkelhals gebrochen. Vielleicht machen Sie sich aus einem anderen Grund Gedanken über das Thema Sturz. Wie bereits angedeutet, kann ein Sturz Verletzungen zur Folge haben. Daher lohnt es sich, über das Thema nachzudenken. Unser Anliegen ist es, Ihnen keine herkömmliche Information an die Hand zu geben. Stattdessen möchten wir eine objektive, auf wissenschaftlichen Untersuchungen basierende Information anbieten. Auf diese Weise können Sie sich Ihre eigene Meinung bilden, wie wichtig das Thema für Sie ist oder werden sollte.

Wir sind ein Team von unabhängigen Wissenschaftlerinnen und wollen Ihnen die neuesten Erkenntnisse der Forschung zum Thema aufzeigen. Besonders wichtig ist uns, dass die Broschüre trotz vieler Informationen verständlich ist. Daher haben wir in Gruppendiskussionen ältere Menschen zu ihrer Meinung über die Broschüre gefragt. Die Ergebnisse der Diskussion haben wir aufgegriffen und in der Broschüre, die Sie jetzt in den Händen halten, berücksichtigt.

Wir stellen uns vor, dass Sie einige Fragen zum Thema Sturz haben. Wir wollen uns auf den folgenden Seiten verschiedenen Fragen zuwenden und versuchen, sie zu beantworten. Folgende Fragen greifen wir auf:

- Wie kann ich erkennen, ob ich ein erhöhtes Sturzrisiko habe?
- Durch welche Maßnahmen kann ich Stürze und Sturz-bedingte Verletzungen vermeiden?
- Welche Maßnahmen sind gut überprüft und welche nicht?
- Wie viele Menschen haben einen Nutzen von der Maßnahme und wie viele haben keinen Nutzen? Oder mit anderen Worten: Wie viele Menschen können tatsächlich einen Sturz vermeiden, wenn sie Sturz-Vorbeugung durchführen?
- Welche Nebenwirkungen haben die Maßnahmen?
- Welche Maßnahmen können also empfohlen werden, und bei welchen ist es unbekannt, ob sie das Sturzrisiko senken?

Wir möchten Ihnen bei der Einschätzung helfen, ob Sie Sturz-gefährdet sind. Auch eine bessere Entscheidung für oder gegen Sturz-vorbeugende Maßnahmen ist durch die Broschüre möglich.

Für wen ist diese Broschüre bestimmt?
Die Broschüre richtet sich an ältere Menschen, die sich über das Risiko und die Vermeidung von Stürzen und Sturz-bedingten Verletzungen informieren möchten.

Angesprochen sind ältere Menschen, die im eigenen Haushalt oder im Betreuten Wohnen leben und ihren Alltag weitgehend selbständig gestalten.

Was wird nicht besprochen?
Leider können wir aus Platzgründen nicht alles in dieser Broschüre ansprechen. Praktische Anleitungen zu Sturz-vorbeugenden Maßnahmen stellen wir nicht vor. Ebenso wenig gehen wir auf einzelne Medikamente zur Sturz-Vorbeugung ein. Auch das Thema Osteoporose (niedrige Knochendichte) behandeln wir nicht. Jedoch finden Sie weiterführende Hinweise im *Kapitel 6*.

Kapitel 6

Welches ist die Grundlage für die Ausführungen?
Bei der Erstellung der Broschüre haben wir die Empfehlungen zur wissensbasierten Patienten- und Verbraucherinformation berücksichtigt. Die Anleitungen der amerikanischen Harvard Universität zum Verfassen von Texten in allgemein verständlicher Sprache wurden ebenfalls aufgegriffen.

Steckelberg, 2005

Harvard School of Public Health, 2008

Kapitel 7

Die Inhalte der Broschüre leiten wir aus wissenschaftlichen Übersichtsarbeiten ab, die den aktuellen Stand der Forschung (Dezember 2008) zusammenfassen.

Tipps zum Lesen der Broschüre

Im Kapitel 2 wird die Häufigkeit von Stürzen und Sturz-bedingten Verletzungen beschrieben. Im Kapitel 3 geht es um die Frage, wie Sie Ihr Sturzrisiko besser einschätzen können. Das Kapitel 4 benennt Maßnahmen zur Vermeidung von Stürzen und Verletzungen und informiert Sie darüber, welchen Nutzen die Maßnahmen haben.

Am Ende der Kapitel 2, 3 und 4 sind die wichtigsten Aussagen des Kapitels zusammengefasst und mit einer grünen Hinterlegung hervorgehoben.

Falls Sie sich entscheiden, Sturz-vorbeugend aktiv zu werden, finden Sie im Kapitel 6 weiterführende Informationen zu praktischen Hilfen sowie Adressen.

Mit den Namen am Rand der Broschüre verweisen wir auf die verwendete Literatur. Diese ist im Anhang aufgelistet. Die verwendeten Fachbegriffe sind gekennzeichnet und an den entsprechenden

Stellen wird am Rand auf das Kapitel 7 „Erklärung verwendeter Fachbegriffe“ verwiesen. Dort können Sie die entsprechenden Begriffe nachschlagen.
Die Broschüre enthält außerdem Abbildungen und Tabellen, auf die im Text hingewiesen wird. Ein Verzeichnis der Abbildungen und Tabellen finden Sie im Anschluss an das Inhaltsverzeichnis.

Wie sicher sind Zahlen aus Studienergebnissen?

Bevor wir uns dem Thema Sturz direkt zuwenden, möchten wir Sie auf die (Un-)Sicherheit von Zahlen aus wissenschaftlichen Studien aufmerksam machen.

Haben Sie sich schon einmal gefragt, ob Sie den Aussagen aus wissenschaftlichen Untersuchungen vertrauen können? Ein kritischer Blick darauf ist nie verkehrt. Denn Studienergebnisse scheinen oft durch die Angabe von konkreten Zahlen „sicher“ zu sein, sind aber in Wirklichkeit mit Unsicherheit behaftet.
Bei Ergebnissen wissenschaftlicher Untersuchungen handelt es sich fast immer um Durchschnittswerte. Für Sie persönlich lassen sich daher keine 100%ig sicheren Aussagen treffen. Auf den nächsten Seiten wollen wir Ihnen aufzeigen, welche wichtigen Hinweise Sie aus den Studien für sich persönlich ableiten können.

Wir hoffen, dass die Broschüre einige Fragen beantworten kann. Schreiben Sie uns gern, wenn Sie Anregungen für uns haben. Post richten Sie bitte an:

Ärztekammer Nordrhein
Frau Priv.-Doz. Dr. med. Dr. P.H. Andrea Icks
Tersteegenstraße 9
40474 Düsseldorf

oder per E-Mail: andrea.icks@aekno.de

Ihre

Andrea Icks

Gabriele Meyer

Sabine Rotzoll

Jutta Genz

2. Stürze und ihre Folgen

2.1 Was ist ein Sturz?

Diese Frage hört sich zunächst banal an. Doch sie ist bedeutsam, damit alle das gleiche meinen, wenn von einem Sturz gesprochen wird. In der Literatur wird der Sturz üblicherweise als ein Ereignis definiert, bei dem ein Mensch unbeabsichtigt auf den Boden oder eine tiefere Ebene gelangt. Auch Ausrutschen und Stolpern mit Landen auf dem Boden sind damit gemeint.

2.2 Wie häufig sind Stürze?

Ungefähr 300 bis 400 von 1000 Senioren über 65 Jahre stürzen mindestens einmal pro Jahr. Anders ausgedrückt: Die Wahrscheinlichkeit innerhalb eines Jahres zu stürzen liegt somit bei 30% bis 40%. Gillespie, 2003
Wer von den 1000 Senioren tatsächlich stürzen wird, kann nicht im Voraus gesagt werden. Bedenken Sie, dass Stürze Sie schon Ihr Leben lang begleiten. Denn Stürze kommen nicht nur im höheren Lebensalter vor. Auch jüngere Menschen stürzen. Dies sehen Sie in der *Abbildung 1, Seite 14*.

Laut einer neueren Studie stürzen von jeweils 1000 Frauen und 1000 Männern in der Altersgruppe von 45 bis 65 Jahren innerhalb von 2 Jahren 250 Frauen und Talbot, 2005

160 Männer. In der Altersgruppe zwischen 20 und 45 Jahren sind es von 1000 Frauen und 1000 Männern 200 Frauen und 170 Männer.

Abb. 1:
Personen mit Sturz in den vergangenen zwei Jahren.*

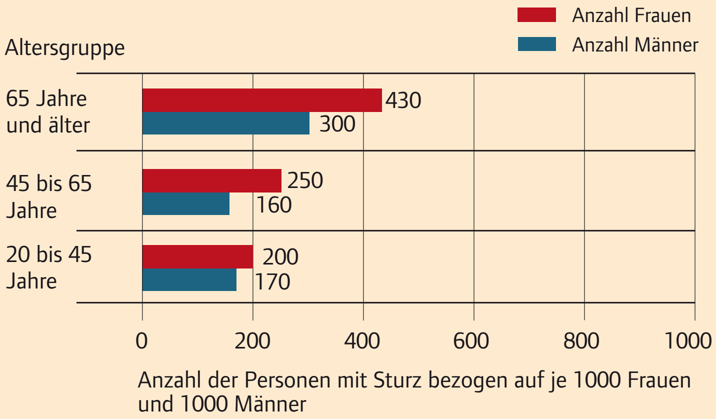

* Hierzu wurden verschiedene Altersgruppen befragt, wie häufig sie in den letzten 2 Jahren gestürzt waren.

2.3 Welche Folgen können Stürze haben?

Gillespie, 2003

Häufig wird einem Sturz nur Bedeutung beigemessen, wenn er zu einer Verletzung führt. Internationale Studien zeigen: Von 1000 Senioren erleiden pro Jahr circa 90 Personen (das heißt: 9%) Verletzungen, die behandelt werden müssen. 30 Personen (das heißt: 3%) erleiden pro Jahr einen Knochenbruch.

Verletzungen treten oft, aber selbstverständlich nicht nur bedingt durch Stürze auf. Die *Tabelle 1* zeigt, wie häufig Unfallverletzungen in Heim und Freizeit sind, die von einem Arzt behandelt werden müssen.

BAuA, 2000/2001

Tabelle 1:
Behandlungsbedürftige Unfallverletzungen zu Hause und in der Freizeit.

Von

1	0	0	0	Personen im Alter von 65 Jahren und älter verletzten sich innerhalb eines Jahres
		3	5	durch einen Sturz,
			9	durch Zusammenstoß mit Gegenstand/Person,
			5	durch einen scharfen oder spitzen Gegenstand,
			4	durch Überbeanspruchung,
			1	durch einen heißen Gegenstand oder Feuer.

Stürze und Sturz-bedingte Verletzungen kommen wie gesagt nicht nur im höheren Lebensalter vor. Jüngere Männer erleiden sogar häufiger Sturz-bedingte Verletzungen als ältere Frauen und Männer. Die Art der Verletzungen unterscheidet sich jedoch in Abhängigkeit vom Lebensalter. So haben jüngere Männer eher „leichtere“ Verletzungen wie Verrenkungen und Schürfwunden, aber seltener Knochenbrüche.

Die *Tabelle 2* veranschaulicht dies.

Tabelle 2:
Anzahl von Sturz-bedingten Verletzungen bei Männern und Frauen.

**Verletzungen** bei je 1000 Personen mit Sturz pro Jahr	Altersgruppe 15–24-jährige		Altersgruppe 75–84-jährige	
	Männer	Frauen	Männer	Frauen
**Verletzungen gesamt,** davon	**343**	**171**	**228**	**249**
Knochenbrüche gesamt	20	5	24	39
davon Oberschenkelhalsbrüche	0	0	4	5
davon Handgelenksbrüche	3	1	1	11
Verrenkungen	79	43	10	19
Kleinere Verletzungen (z.B. Prellungen, Schürfwunden)	228	109	154	148

Nach Van Weel, 1995: Bevölkerungsbezogene Erhebung, Niederlande.

2.4 Wie häufig sind Oberschenkelhalsbrüche?

Dieser Frage wenden wir uns intensiver zu,
Kapitel 7 denn Oberschenkelhalsbrüche („Hüftfrakturen“ oder auch „Hüftbrüche“ genannt) gehören zu den folgenreichsten Sturz-bedingten Verletzungen. In

Deutschland ereignen sich pro Jahr bei 1000 Senioren im Alter von 65 Jahren und älter insgesamt 7 Oberschenkelhalsbrüche (das heißt: 0,7%). Die Mehrzahl ist durch einen Sturz bedingt. Icks, 2008
Die Anzahl der Oberschenkelhalsbrüche steigt mit dem Alter bei Frauen mehr an als bei Männern. Die *Abbildung 2* veranschaulicht dies.

Abb. 2:
Häufigkeit von Oberschenkelhalsbrüchen bei über 65-Jährigen.

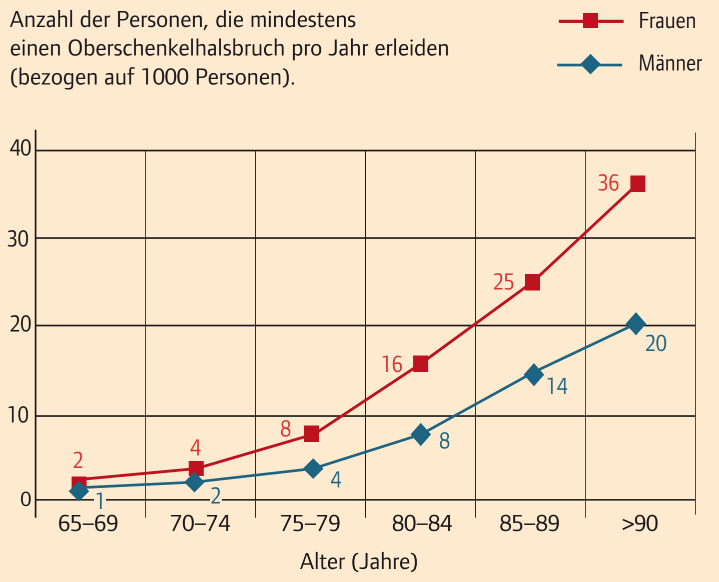

Nach Icks et al., 2008.

Von 1000 Frauen im Alter von über 90 Jahren werden demnach ungefähr 36 Frauen (das heißt: 3,6%) innerhalb eines Jahres einen Oberschenkelhalsbruch erleiden, bei den Männern im Alter von über 90 Jahren sind es ungefähr 20 Männer (das heißt: 2,0%).

2.5 Welche Folgen haben Oberschenkelhalsbrüche?

Pientka, 1999

Von 1000 selbständigen Personen, die einen Oberschenkelhalsbruch erleiden, kann sich nach dem Bruch die Hälfte (50%) nicht mehr so gut bewegen wie vorher. 100 bis 200 von 1000 Personen (das heißt: 10% bis 20%) werden dauerhaft pflegebedürftig.

Zusammenfassung:

Stürze treten in jedem Alter auf. Senioren stürzen häufiger als jüngere Menschen, und die Folgen sind in der Regel schwerwiegender. Von 1000 Senioren im Alter von über 65 Jahren stürzen im Jahr zwischen 300 und 400 Personen (das heißt: 30% bis 40%). Eine behandlungsbedürftige Verletzung erleiden von 1000 Personen circa 90 Personen (das heißt: 9%) pro Jahr. Einen Oberschenkelhalsbruch erleiden in einem Jahr von 1000 Personen circa 7 Personen (das heißt: 0,7%).

3. Erkennen des Sturzrisikos

3.1 Wie kann das individuelle Sturzrisiko bestimmt werden?

Sie haben im letzten Kapitel gelesen, wie häufig Stürze bei Senioren vorkommen. Jetzt möchten Sie vielleicht aktiv werden und überlegen, wie Sie Stürze vermeiden können. Dann geht es Ihnen ähnlich wie Frau Möller, die wir Ihnen jetzt vorstellen möchten.

Frau Möller ist 71 Jahre alt. Bei der wöchentlichen Chorprobe erfährt sie, dass Frau Siebert, eine gute Bekannte, einen Tag zuvor in die Unfallambulanz eingeliefert wurde. Frau Siebert ist auf der Dachbodentreppe gestolpert, gestürzt und hat sich das Handgelenk gebrochen. Frau Möller besucht ihre Bekannte am nächsten Tag. Frau Siebert freut sich über den Besuch von Frau Möller und erzählt ihr, wie es zu dem Sturz gekommen ist. Frau Möller macht sich in den nächsten Tagen immer wieder Gedanken darüber, ob auch sie stürzen könnte. Sie möchte vorbeugen. Sie hat gehört, dass Bewegungsübungen helfen sollen, Stürze zu vermeiden. Obwohl sie noch nie richtig Motivation hatte, Sport zu treiben, wäre sie eventuell bereit, an einer Sportgruppe teilzunehmen.

Eine Woche darauf trifft Frau Möller Frau Siebert bei der Chorprobe wieder. Sie erzählt ihrer Bekannten vom Sportkurs. Frau Siebert macht ihr den Vorschlag, erst einmal zu überprüfen, wie sturzgefährdet sie ist.

Der Vorschlag von Frau Siebert erscheint logisch. Bevor Sie Sturz-vorbeugende Maßnahmen durchführen, ist es sinnvoll zu prüfen, ob Sie ein durchschnittliches oder ein erhöhtes Sturzrisiko haben. Denn es gibt Sturz-vorbeugende Maßnahmen, die nur Menschen mit einem erhöhten Sturzrisiko nützen. Darauf gehen wir in *Kapitel 4* ein. Hier erst einmal zum Risiko: Mit der Angabe des Risikos wird die Wahrscheinlichkeit für einen Sturz oder eine Sturz-bedingte Verletzung angegeben. Ein Risiko zu stürzen ist jedoch nicht automatisch gleichzusetzen mit der Gewissheit zu stürzen. Sie können also nicht vorhersagen, ob Sie tatsächlich stürzen werden, aber Sie können die Wahrscheinlichkeit dafür abschätzen.

3.2 Wie hoch ist das Risiko zu stürzen?

Wie bereits im Kapitel 2.2 beschrieben, liegt die Wahrscheinlichkeit innerhalb eines Jahres zu stürzen für Personen im Alter über 65 Jahren bei 30% bis 40%. Diese Sturzwahrscheinlichkeit ist ein

durchschnittliches Risiko. Hinzu kommen verschiedene Faktoren, mit denen sich das Risiko verändern kann.

Diese Merkmale und Bedingungen werden „Risikofaktoren oder Risikoindikatoren“ genannt.

Kapitel 7

3.3 Welche Risikofaktoren und Risikoindikatoren gibt es?

In der Literatur werden viele mögliche Faktoren als Risiko*faktoren* oder Risiko*indikatoren* für Stürze beschrieben. Risiko*faktoren* sind solche, die als Ursache für einen Sturz angesehen werden können. Risiko*indikatoren* sind Faktoren, die ein erhöhtes Sturzrisiko anzeigen (zum Beispiel das erhöhte Alter).

Für viele in der Literatur beschriebene Faktoren ist nicht eindeutig nachgewiesen, dass sie etwas mit einem Sturzereignis zu tun haben.

Die Frage ist daher: Welche Risikofaktoren oder Risikoindikatoren wurden bislang tatsächlich in wissenschaftlichen Studien bestätigt?

Wie schon erwähnt, sind dies Alter und Geschlecht: Frauen stürzen im höheren Alter bis zu dreimal

häufiger als Männer. Ältere Menschen haben ein höheres Risiko als jüngere Menschen.
Von 1000 Personen im Alter von über 80 Jahren stürzen etwa 500 mindestens einmal pro Jahr.

Auch ein Sturz in der Vergangenheit und eine Beeinträchtigung von Gang und Gleichgewicht sind als Risikofaktoren bestätigt.

Ganz, 2007 Weniger gut als Risikofaktoren belegt sind die Einnahme bestimmter Medikamente, die Beeinträchtigung von Alltagsaktivitäten (zum Beispiel Probleme bei der Fortbewegung), ein schlechteres Sehvermögen und eine beginnende Demenz.

Sie können Ihr Sturzrisiko mit Ihrem Hausarzt oder Ihrer Hausärztin besprechen. Er oder sie kann Ihnen eine individuelle Untersuchung anbieten. Dabei werden anhand von Tests die Risikofaktoren für Stürze erhoben. Dazu gehört eine Beurteilung Ihrer Gangsicherheit, Ihrer Muskelkraft und Ihres Körpergleichgewichts. Sie können Ihr eigenes Risiko aber auch selbst abschätzen. Dazu können Sie die Risikofaktoren auflisten, die für Sie zutreffen. Das

weitere Vorgehen beschreiben wir in den folgenden Abschnitten.

3.4 Wie hoch ist Ihr persönliches Risiko zu stürzen?

Es ist nicht möglich, Ihr ganz genaues Risiko zu bestimmen. Denn neben den untersuchten Risikofaktoren und Risikoindikatoren gibt es weitere Faktoren, die das Sturzrisiko beeinflussen. Außerdem kann aus keiner Studie die Höhe des Sturzrisikos abgelesen werden, wenn mehrere Risikofaktoren gleichzeitig auftreten. Trotzdem können Sie Ihre Wahrscheinlichkeit zu stürzen zumindest abschätzen. Wie bereits in Kapitel 2.2 beschrieben, liegt die Wahrscheinlichkeit innerhalb des nächsten Jahres zu stürzen für Personen im Alter über 65 Jahre im Durchschnitt bei 30% bis 40%. Je nachdem, welche persönlichen Risikofaktoren vorliegen, ist die Wahrscheinlichkeit zu stürzen verändert. Wir zeigen hier nur die Erhöhung des Risikos, da es in dieser Broschüre um erhöhte Risiken und Vermeidung geht. Die nachfolgende Tabelle zeigt Ihnen diese Risikofaktoren und die Änderung des Sturzrisikos.

3. Erkennen des Sturzrisikos

Tabelle 3: **Risikofaktoren und Wahrscheinlichkeit in 12 Monaten zu stürzen.**

**Risikofaktor/-indikator**	**Genauere Umschreibung**	**Wahrscheinlichkeits-verhältnisse** (Unsicherheitsbereich)	**Individuelles Sturzrisiko*** (Unsicherheitsbereich)
Sturz in der Vergangenheit	Sturz im letzten Jahr	**2,8** (2,1 bis 3,8)	**55%** (47% bis 62%)
	Sturz im letzten Monat	**3,8** (2,2 bis 6,4)	**62%** (49% bis 73%)
Beeinträchtigung von Gang und Gleichgewicht	Leichte Gleichgewichtsprobleme (Frauen)	**2,0** (1,5 bis 2,7)	**46%** (39% bis 54%)
	Leichte Gleichgewichtsprobleme (Männer)	**2,3** (1,6 bis 3.3)	**50%** (41% bis 59%)
	Gang- und Gleichgewichtsprobleme	**2,8** (1,8 bis 4,4)	**55%** (44% bis 65%)
	Entzündliche Erkrankung des Kniegelenks (Frauen)	**1,3** (1,1 bis 1,6)	**36%** (32% bis 41%)
	Entzündliche Erkrankung des Kniegelenks (Männer)	**1,6** (1,3 bis 2,0)	**41%** (36% bis 46%)
	Selbsteinschätzung der Befragten	**1,8** (1,5 bis 2,2)	**44%** (39% bis 49%)
Medikamente	Einnahme von 4 oder mehr Medikamenten	**1,9** (1,4 bis 2,5)	**45%** (38% bis 52%)
	Einnahme von Medikamenten, die auf das Gemüt und das Bewusstsein wirken	**1,7** (1,3 bis 2,2)	**42%** (36% bis 49%)
	Einnahme von bestimmten Medikamenten für das Gemüt, gegen Depression oder Einnahme von Schlaf- und Beruhigungsmitteln	**27** (3,6 bis 207)	**92%** (61% bis 99%)
Beeinträchtigung der Alltagsfähigkeiten	Probleme beim Aufstehen aus einem Stuhl	**4,3** (2,3 bis 7,9)	**65%** (50% bis 77%)
	Vermehrte Bettlägerigkeit im letzten Monat	**3,7** (1,6 bis 8,6)	**61%** (41% bis 79%)
	Verschlechterung des Gesundheitszustandes im letzten Jahr	**2,0** (1,5 bis 2,9)	**46%** (39% bis 55%)
	Mehrere Alltagsbeeinträchtigungen zusammen	**1,9** (1,4 bis 2,6)	**45%** (38% bis 53%)
Beeinträchtigung der geistigen Fähigkeit	Diagnose Demenz	**17** (1,9 bis 149)	**88%** (45% bis 98%)
	Gedächtnisprobleme	**4,2** (1,9 bis 9,6)	**64%** (45% bis 80%)

Erläuterung der Tabelle auf *Seite 26*

* Die Berechnungen des individuellen Sturzrisikos gehen von einem durchschnittlichen Sturzrisiko von 30% aus.
Das Risiko bei Vorliegen des jeweiligen Risikofaktors wurde anhand einer Umrechnungsformel berechnet.

Ganz, 2007 Die *Tabelle 3 auf den Seiten 24 und 25* führt in der linken Spalte verschiedene Risikofaktoren auf. Diese Risikofaktoren werden in der mittleren Spalte noch weiter erklärt und beschrieben. In der Spalte hinter den Risikofaktoren steht eine fett gedruckte Zahl, die

Kapitel 7 als Wahrscheinlichkeitsverhältnis bezeichnet wird. Das Wahrscheinlichkeitsverhältnis zeigt, wie viel größer das Sturzrisiko bei einer Person mit dem Risikofaktor im Vergleich zu einer Person ohne diesen Risikofaktor ist. Zum Beispiel: Eine ältere Person mit einem Sturz im letzten Monat hat eine 3,8mal höhere Wahrscheinlichkeit zukünftig zu stürzen als eine ältere Person, die im letzten Monat nicht gestürzt ist. Dabei stellt die fett gedruckte Zahl (das Wahrscheinlichkeitsverhältnis) einen durchschnittlichen Wert dar. Zu den fett gedruckten Zahlen werden in Klammern jeweils eine kleinere und eine größere Zahl angegeben, das ist der

Kapitel 7 so genannte Unsicherheitsbereich. Die genaue Wahrscheinlichkeit oder das genaue Wahrscheinlichkeitsverhältnis kann mittels Studien nicht exakt bestimmt werden, da Studienergebnisse natürlichen Zufallsschwankungen unterliegen. Daher ist in der Tabelle auch der so genannte Unsicherheitsbereich angegeben. Diese Zahlen sind die Faktoren mittels derer das persönliche Risiko errechnet werden kann.

In der helleren Spalte ganz rechts ist das Sturzrisiko bei Vorliegen der verschiedenen Risikofaktoren angegeben. Auch hier stellt die zuerst genannte Prozentzahl einen Durchschnittswert dar. Die Prozentwerte in den Klammern zeigen den Bereich auf, indem das Sturzrisiko liegen kann. In diesem Bereich ist also angegeben, wie wahrscheinlich eine Person bei Vorliegen des Risikofaktors im nächsten Jahr stürzen wird.

Frau Möller hat ihr „persönliches Sturzrisiko" ermittelt.
So ging sie vor:
Einige Risikofaktoren konnte sie ausschließen:

- *„Sturzereignis in der Vergangenheit", da sie bisher nicht gestürzt ist.*
- *„Medikamente", da sie nur ein Medikament gegen zu hohe Blutfettwerte einnimmt.*
- *„Beeinträchtigungen der Alltagsaktivitäten", da sie alles noch alleine erledigt.*
- *„Demenz" liegt bei Frau Möller auch nicht vor.*

Allerdings hat Frau Möller in der letzten Zeit immer wieder beim Gehen und Aufstehen vom Stuhl leichte Gleichgewichtsprobleme gehabt. Da sie manchmal beim Gehen schwankte, musste sie sich öfter bei ihrem Mann einhaken.

In der *Tabelle 3* steht in der mittleren Spalte bei dem Punkt „leichte Gleichgewichtsprobleme (Frauen)“ die fett gedruckte Zahl **2,0** und in Klammern (1,5 bis 2,7). In der Spalte daneben Steht die Zahl **46%** und in Klammern (39% bis 54%). Somit würde das persönliche Sturzrisiko für einen Sturz im nächsten Jahr für Frau Möller bei durchschnittlich 46% liegen. *Frau Möller schaut sich auch die Angaben in den Klammern an. Ihr Risiko für einen Sturz im nächsten Jahr liegt demnach im Bereich zwischen 39% und 54%. Die Abbildung 3 veranschaulicht den Weg, mit dem man von der mittleren Spalte zu der Spalte daneben gelangt. Der hellgrau hinterlegte Bereich ist derjenige Bereich, in dem das Sturzrisiko von Frau Möller liegt.* Das Risiko kann auf dieser Abbildung abgelesen werden: Links ist das durchschnittliche Sturzrisiko für Personen im Alter von über 65 Jahren in Höhe von 30% angegeben, in der Mitte befindet sich der Faktor, mit dem der Risikofaktor zu Buche schlägt. Auf der rechten Seite der Abbildung kommt schließlich das Risiko bei Vorliegen des Risikofaktors heraus.

3.5 Welche Merkmale sind mit einem erhöhten Risiko für Oberschenkelhalsbrüche verbunden?

Im vorherigen Kapitel haben wir Ihnen erläutert, wie

Abb. 3:
Skala zum Ablesen der Sturzwahrscheinlichkeit innerhalb des nächsten Jahres.

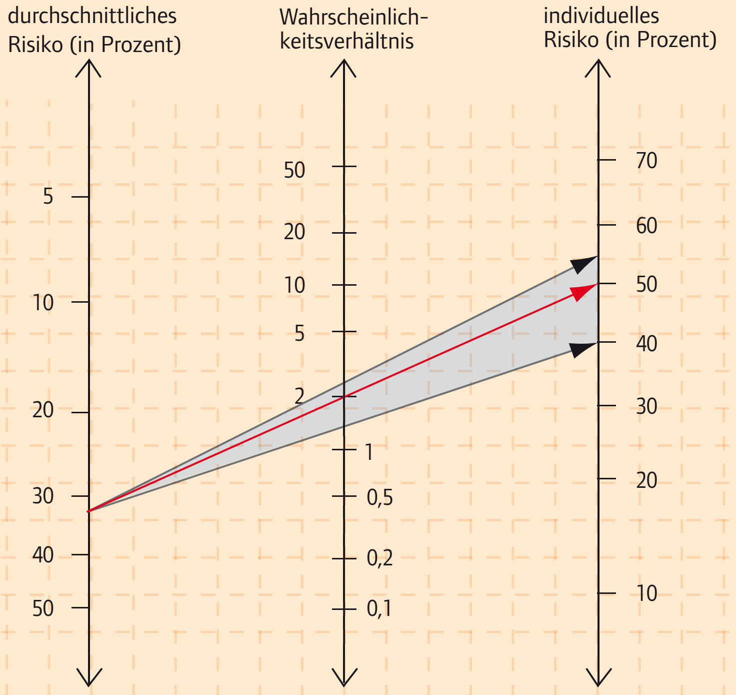

Sie Ihr „persönliches Risiko“ für einen Sturz abschätzen können. Diese Zahlen geben die Wahrscheinlichkeiten für einen Sturz an, bei dem die Folgen ganz unterschiedlich sein können.

Wie hoch ist das Risiko, in einem Jahr einen Oberschenkelhalsbruch zu erleiden? Wie im Kapitel 2.4 beschrieben, beträgt das Risiko, einen Oberschenkelhalsbruch zu erleiden, für Personen im Alter von über 65 Jahren innerhalb eines Jahres 0,7%. Sie können in der *Abbildung 2 auf Seite 17* dieses Risiko für Ihr Alter, unterteilt nach Männern und Frauen, ablesen. Zum Beispiel liegt das Risiko für eine Frau im Alter von 80 bis 84 Jahren bei rund 15%.

Nguyen, 2007

Weitere Merkmale, die das Risiko für einen Oberschenkelhalsbruch erhöhen, beschreibt eine neuere Forschungsarbeit. Diese stellt die Ergebnisse einer Langzeitstudie über 15 Jahre mit fast 2000 älteren Menschen dar.

Demnach erhöht sich das Risiko für einen Oberschenkelhalsbruch bei Senioren

- die in den letzten fünf Jahren schon einmal einen Knochenbruch erlitten haben,
- die im letzten Jahr einen Sturz erlitten haben,
- bei denen die Knochendichtemessung eine verminderte Knochendichte am Oberschenkelknochen festgestellt hat, (Kapitel 7)
- bei denen Störungen des Gleichgewichts vorliegen,

3. Erkennen des Sturzrisikos

- die ein geringes Körpergewicht haben, und
- die klein sind.

Zusammenfassung:

Haben Sie ein erhöhtes Sturzrisiko?
Bevor Sie Sturz-vorbeugende Maßnahmen ergreifen, erscheint es sinnvoll zu prüfen, wie hoch Ihr Sturzrisiko ist. Generell stürzen Frauen im Alter häufiger als Männer. Das Risiko zu stürzen ist für ältere Menschen höher als für jüngere. Das durchschnittliche Sturzrisiko für Personen im Alter von über 65 Jahren liegt zwischen 30% und 40%. Es erhöht sich durch hinzukommende persönliche Sturzrisikofaktoren. Überprüfen Sie, welche Risikofaktoren für einen Sturz aus der *Tabelle 3* für Sie zutreffen. Sie können somit Ihr persönliches Sturzrisiko für ein Jahr abschätzen. Ein erhöhtes Sturzrisiko zu haben, bedeutet jedoch nicht, dass Sie tatsächlich stürzen werden. Außerdem müssen Stürze nicht immer mit einem Knochenbruch oder einer anderen schweren Folge einhergehen.

Das Risiko für einen Oberschenkelhalsbruch ist bei Frauen größer als bei Männern und steigt mit dem Alter an.
Es erhöht sich bei Vorliegen bestimmter Risikofaktoren.

4. Maßnahmen zur Vermeidung von Stürzen und Sturzfolgen

4.1 Welche Maßnahmen zur Vermeidung von Stürzen gibt es?

Viele verschiedene Maßnahmen zur Vermeidung von Stürzen wurden bisher untersucht. Zu den häufigsten zählen:

- **Wohnraumanpassung:**
die Anpassung und Veränderung des Wohnraums, um die Sicherheit zu erhöhen.

- **Bewegungsübungen:**
unterschiedliche Übungen, die meistens Kraft- und Gleichgewichtstraining enthalten.

- **Augenprüfung:**
Überprüfung und gegebenenfalls Ausgleich beeinträchtigter Sehleistung.

- **Medikamentenprüfung:**
eine Überprüfung und gegebenenfalls Anpassung der eingenommenen Medikamente.

- **Hüftprotektoren:**
Tragen von Hüftprotektoren zur Vermeidung eines Oberschenkelhalsbruches im Fall eines Sturzes.

Diese Maßnahmen werden wir Ihnen nun näher vorstellen und danach auf ihren Nutzen eingehen.

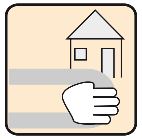

Wohnraumanpassung: Verbesserung der Sicherheit in der Wohnung.

Zu diesen Maßnahmen zählen Veränderungen in der Wohnung, beispielsweise die Verbesserung der Beleuchtung, das Anbringen von rutschfesten Matten, Handläufen an den Treppen und Haltegriffen im Bad. Aber auch eine Anleitung für ein sicheres Verhalten in der Wohnung gehört dazu. Das Tragen von Rutsch-hemmenden Socken oder die Verwendung von Gehhilfen wird oft empfohlen. Hinweise zu Broschüren und Ansprechpartnern finden Sie im *Kapitel 6*.

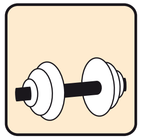

Bewegungsübungen: unterschiedliche Übungen, die meistens Kraft- und Gleichgewichtstraining enthalten.

Meistens enthalten Bewegungsübungen ein Training von Kraft und/oder Gleichgewicht. Die Übungen können einzeln oder in Gruppen durchgeführt werden. Das Krafttraining beinhaltet Übungen für Arme, Beine und den Oberkörper. Beim Gleichgewichtstraining werden sicheres Stehen und Gehen, das Drehen des Körpers

und das Halten des Gleichgewichts in „wackeligen“ Situationen trainiert. Die Übungen werden mit einfachen, auch im Alltag einsetzbaren Kleingeräten durchgeführt. Für das Krafttraining werden häufig Handgewichte (Hanteln) und Gewichtsmanschetten für die Beine verwendet, für das Gleichgewichtstraining Seile, Luftballons oder kleine Bälle. Auch Bewegungsübungen
Kapitel 7
aus dem chinesischen Tai Chi werden genutzt. Hinweise zu Broschüren und Übungen finden Sie im *Kapitel 6*.

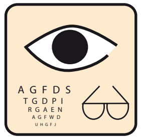

Augenprüfung: Überprüfung und gegebenenfalls Ausgleich beeinträchtigter Sehleistung.

Die Sehleistung wird bei einer augenärztlichen Untersuchung überprüft. Neben einer eingeschränkten Sehschärfe durch Alterssichtigkeit kann zum Beispiel eine Trübung der Linse (grauer Star) die Sehleistung beeinträchtigen. Viele Einschränkungen der Sehleistung können korrigiert werden.

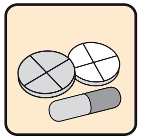

Medikamentenprüfung: eine Überprüfung von Medikamenten, die das Sturzrisiko erhöhen.

Einige Medikamente, zum Beispiel bestimmte Beruhigungs- oder Schlafmittel, können das Sturzrisiko

4. Maßnahmen zur Vermeidung von Stürzen und Sturzfolgen

erhöhen. Sie können mit Ihrem Hausarzt besprechen, ob die von Ihnen eingenommenen Medikamente Ihr Sturzrisiko erhöhen.

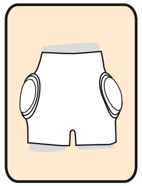

Hüftprotektoren: Tragen von Hüftprotektoren zur Vermeidung eines Oberschenkelhalsbruches im Fall eines Sturzes.

Ein mechanisches Hilfsmittel ist der Hüftprotektor. Bei einem Sturz puffert er die auf die Hüftregion einwirkende Kraft ab. Es handelt sich um Schalen oder Polster, die meistens in eine Hüftschutzhose eingenäht sind. Hüftprotektoren vermeiden keine Stürze, sondern sollen im Falle eines Sturzes den Bruch des Oberschenkelhalses verhindern.

4.2 Welchen Nutzen haben Maßnahmen zur Vermeidung von Stürzen?

Sturz-vorbeugende Maßnahmen wurden in Studien teilweise einzeln, oft auch in Kombination untersucht. So wurde zum Beispiel eine individuelle Beratung zu Sturzrisiken in Kombination mit Bewegungsübungen und einer Korrektur des Sehens untersucht. Außerdem wurden die Maßnahmen teilweise bei allen Senioren eingesetzt, teilweise aber nur bei Senioren, die ein

erhöhtes Sturzrisiko haben (also beispielsweise bei denjenigen, die bereits gestürzt sind).

Die international vorliegenden Studien zur Sturz-Vorbeugung wurden zusammenfassend in einer systematischen Übersichtsarbeit geprüft. Übersichtsarbeiten liefern die besten Hinweise zum Nutzen von Maßnahmen. Wir verwenden eine ausführliche Arbeit von Gillespie, die 2003 entstanden ist. Eine neuere ausführliche Übersichtsarbeit liegt bislang nicht vor. Seither wurden eine Reihe weiterer Studien durchgeführt. Diese haben wir ebenfalls gesichtet und kommen zu dem Schluss, dass die Ergebnisse der Übersichtsarbeit aus dem Jahr 2003 auch heute noch im Wesentlichen zutreffen. Änderungen durch neuere Ergebnisse haben wir eingefügt.

Kapitel 7

Gillespie, 2003

Meyer, 2009

Wir beschreiben zunächst exemplarisch den Nutzen einer ausgewählten Maßnahme.

Gillespie, 2003

In der genannten Übersichtsarbeit wurden drei Studien zusammengetragen, die in vergleichbarer Weise den Nutzen einer Wohnraumberatung zur Vorbeugung von Stürzen untersucht haben.

Die Programme richteten sich an Senioren aus Deutschland, Frankreich und Australien, die zu Hause lebten und in der Vergangenheit schon einmal gestürzt waren, also ein erhöhtes Sturzrisiko hatten. Bei jedem Studienteilnehmer wurde ein Hausbesuch durchgeführt. Dabei wurden die Personen beraten, wie sie ihre Wohnung sicherer gestalten können. Teilweise erfolgte ein zweiter Besuch oder ein nachfolgendes Telefonat. Es nahmen 374 Senioren an der Studie teil. In der Gruppe mit dem Programm stürzten 77 von 186 Senioren. In der Gruppe ohne Programm stürzten 118 von 188 Senioren. Hochgerechnet auf jeweils 1000 Senioren ergibt sich: Von 1000 Senioren, die das Programm erhalten, stürzen 414 in einem Jahr. Im Vergleich dazu: Von 1000 Senioren, die das Programm nicht erhalten, stürzen 628 in einem Jahr. Somit haben 214 (628 minus 414) von 1000 Senioren einen Vorteil durch das Programm, da sie dadurch nicht stürzen. Aber: 786 von 1000 Senioren haben keinen Vorteil, da sie entweder trotz des Programms stürzen (414 Senioren) oder sowieso nicht stürzen (372 Senioren).

An diesem Beispiel wird deutlich, dass Sturz-vorbeugende Maßnahmen nur bedingt nützlich sind. *Abbildung 4* veranschaulicht das beschriebene Beispiel.

Abb. 4: **Nutzen von Wohnraumberatung.**

Dargestellt sind 1000 Senioren, wobei 414 Senioren trotz des Programmes gestürzt sind (=), 372 Senioren mit oder ohne das Programm nicht gestürzt sind (=) und 214 Senioren einen Nutzen haben, da sie durch das Programm nicht gestürzt sind (=).

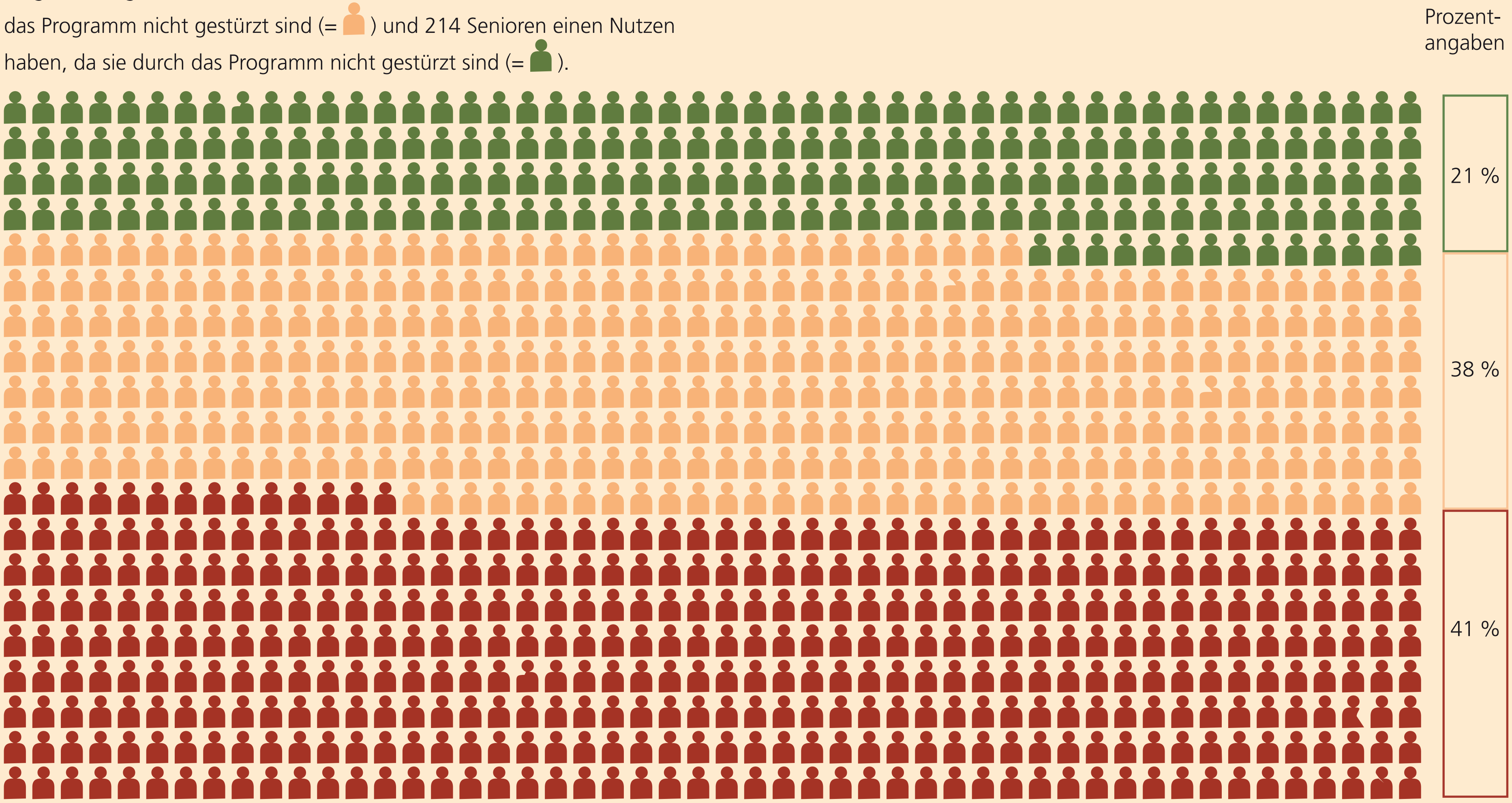

Der Nutzen kann auch anders ausgedrückt werden. Und zwar durch die Angabe, wie viele Senioren an einer Sturz-vorbeugenden Maßnahme oder an einem Programm teilnehmen müssen, damit eine Person vor dem Stürzen bewahrt wird. Dabei ist die Maßnahme umso vorteilhafter, je niedriger die Anzahl der Personen ist, die teilnehmen muss. In der *Tabelle 4* führen wir die Maßnahmen auf, für die nach der vorliegenden Literatur ein Nutzen belegt ist. Wir zeigen dabei die durchschnittliche Anzahl der Personen, die an der Maßnahme teilnehmen müssen, um eine Person vor dem Stürzen zu bewahren.

Sherrington, 2008

Eine aktuelle Übersichtsarbeit hat bestätigt, dass Bewegungsübungen noch nützlicher sind, wenn sie:

- 2-mal wöchentlich über einen längeren Zeitraum (hier: 25 Wochen),
- zusammen mit Gleichgewichtsübungen durchgeführt werden und
- kein Walking-Programm (schnelles Gehen) beinhalten.

Campbell, 2007

Es gibt viele Möglichkeiten, Maßnahmen der Sturz-vermeidung zu kombinieren. Mehrere Maßnahmen gleichzeitig anzuwenden ist jedoch nicht zwangsläufig nützlicher als eine einzelne Maßnahme durchzuführen.

Tabelle 4:
Maßnahmen mit belegtem Nutzen zur Vermeidung von Stürzen.

**Maßnahme** (Anzahl der Studien)	**Anzahl Senioren, die an einem sturz-vorbeugenden Programm teilnehmen müssen, um eine Person vor dem Stürzen zu bewahren** (Unsicherheitsbereich)
Übung von Kraft und Gleichgewicht bei Senioren mit erhöhtem Sturzrisiko (3 Studien)	**11** (6–120)
Verbesserung der Sicherheit in der Wohnung	
■ bei Senioren die bereits gestürzt waren (3 Studien)	**5** (3–9)
■ bei Senioren mit oder ohne früheren Sturz (3 Studien)	**14** (8–75)
Bewegungsübungen und Korrektur der Sehleistung und Verbesserung der Sicherheit in der Wohnung (1 Studie)	**6** (4–27)
Bewegungsübungen und Korrektur der Sehleistung (1 Studie)	**7** (4–30)
Untersuchung des Sturzrisikos und Programm aus verschiedenen Maßnahmen*	
■ bei einer gemischten Gruppe von Senioren (4 Studien)	**11** (8–23)
■ bei Senioren mit Sturz in der Vergangenheit oder individuell bestimmtem Sturzrisiko (5 Studien)	**13** (8–53)

*Kombination aus Bewegungsübungen, Veränderungen in der Wohnung und/oder Überprüfung der Medikamente.

Nach Gillespie, 2003

Die *Tabelle 4* zeigt, dass Maßnahmen teils auch bei Senioren nützlich sind, die kein erhöhtes Sturzrisiko haben. Die vorliegenden wissenschaftlichen Ergebnisse sprechen aber dafür, dass Maßnahmen zur Sturzvermeidung erfolgreicher sind, wenn die teilnehmenden Senioren ein erhöhtes Sturzrisiko haben, also beispielsweise bereits gestürzt sind.

4.3 Für welche Maßnahmen fehlt ein Nachweis der Sturz-vorbeugenden Wirkung?

Für folgende Einzelmaßnahmen und Kombinationen aus Einzelmaßnahmen konnte bisher kein Nutzen belegt werden:

Einzelmaßnahmen:

Gillespie, 2003

- Kraft- und Gleichgewichtsübungen bei Senioren ohne erhöhtes Sturzrisiko, alleinige Kraftübungen bei Senioren mit einem erhöhten Sturzrisiko,
- Überprüfung der eingenommenen Medikamente,
- Verbesserung der Sicherheit der Wohnung bei Senioren, die noch nicht gestürzt sind,

Parker, 2006

- Angebot von Hüftprotektoren zur Vermeidung von Oberschenkelhalsbrüchen bei Senioren, die im eigenen Haushalt leben.

4. Maßnahmen zur Vermeidung von Stürzen und Sturzfolgen

Kombinierte Maßnahmen:

Gillespie, 2003

- Bewegungsübungen und Verbesserung der Sicherheit der Wohnung,
- Verbesserung der Sicherheit der Wohnung kombiniert mit einer Überprüfung der eingenommenen Medikamente oder mit Beratung zur Sturz-Vorbeugung,

Elley, 2008
Hendriks, 2008

- Ermittlung der Sturzrisikofaktoren (individuelle und Wohnraum-bedingte) und Vorschläge, den Hausarzt, Optiker, die Fußpflege, Krankengymnastik oder Beschäftigungstherapie aufzusuchen (in einer Studie zusätzlich: Bewegungsübungen zu Hause).

Latham, 2003

Wenn für eine Maßnahme bislang noch kein Nutzen belegt ist, kann dieses vielleicht in Zukunft noch geschehen. Viele Sturz-vorbeugende Maßnahmen wurden bislang in kleinen Studien mit wenigen Teilnehmern untersucht. Solche kleinen Studien können jedoch häufig keine aussagekräftigen Ergebnisse erbringen. Maßnahmen nach „gesundem Menschenverstand“, wie zum Beispiel, nicht auf wackelige Leitern zu steigen oder bei Schnee und Eis rutschfeste Schuhe zu tragen, werden nicht in wissenschaftlichen Studien untersucht. Diese Maßnahmen sind selbstverständlich vernünftig

und leicht umzusetzen. Sie könnten für sich selbst überlegen, wie Sie durch solche „vernünftigen Maßnahmen“ Ihr persönliches Sturzrisiko beeinflussen können.

4.4 Welchen Nutzen haben Maßnahmen zur Vermeidung von Sturz-bedingten Verletzungen?

Gillespie, 2003

In der Übersichtsarbeit wurde ein Teil der bereits beschriebenen nützlichen Maßnahmen daraufhin überprüft, ob sie außer dem Risiko zu stürzen auch das Risiko für Sturz-bedingte Verletzungen vermindern können.

Lediglich für 2 Maßnahmen konnte dieser Nutzen belegt werden:

- individuell ausgerichtete Übungen von Muskelkraft und Gleichgewicht bei Senioren mit erhöhtem Sturzrisiko (5 Studien): hier mussten 9 Personen (Unsicherheitsbereich: 6 bis 31) an der Maßnahme teilnehmen, um bei einer Person eine Sturz-bedingte Verletzung zu vermeiden,
- ausführliche Untersuchung des Sturzrisikos mit anschließendem Angebot individuell angepasster Maßnahmen (Bewegungsübungen, Veränderung der Wohnung und/oder Überprüfung der Medikamente)

bei Senioren mit Stürzen in der Vergangenheit oder hohem Sturzrisiko (1 Studie): hier mussten 22 Personen (Unsicherheitsbereich: 12 bis 103) an der Maßnahme teilnehmen, um eine Person vor einer Sturz-bedingten Verletzung zu bewahren.

Für die anderen genannten Sturz-vorbeugenden Maßnahmen konnte bisher nicht belegt werden, dass sie das Risiko für Sturz-bedingte Verletzungen senken.

Gillespie, 2003

4.5 Eigene Vorlieben berücksichtigen?

Die beschriebenen Maßnahmen zur Vermeidung von Stürzen können sich unabhängig von ihrem Sturz-vorbeugenden Nutzen auf das persönliche Wohlbefinden auswirken. Vielleicht fühlen Sie sich durch Haltegriffe im Bad Ihrer Wohnung sicherer. Möglich ist natürlich auch, dass eine bestimmte Maßnahme nicht Ihren Vorlieben entspricht, da Sie zum Beispiel wie Frau Möller nicht gern Sport treiben. Sie werden Ihre Vorlieben und Abneigungen berücksichtigen, wenn Sie sich für oder gegen Maßnahmen entscheiden.

Doch nun noch mal zurück zu Frau Möller.
Bei Gleichgewichtsstörungen kann ein individuell

ausgerichtetes Gleichgewichtstraining zur Vorbeugung von Stürzen sinnvoll sein. Frau Möller kann sich mit einem Gleichgewichtstraining mehr anfreunden als mit der Sportgruppe. Sie möchte sich jetzt informieren, wie sie die Übungen durchführen kann.

Falls Sie sich persönlich auch informieren möchten, finden Sie Adressen und Hinweise im Kapitel 6.

4.6 Welche Nebenwirkungen haben Sturz-vorbeugende Maßnahmen?

Zu dieser Frage gibt es kaum aussagefähige Ergebnisse, da Nebenwirkungen in den Studien nicht ausreichend überprüft wurden. Eine ältere Studie zeigte, dass Seniorinnen mit bereits erlittenen Knochenbrüchen durch schnelles Gehen (Walking) ein erhöhtes Sturzrisiko haben. Eine andere Studie fand ein gesteigertes Risiko für Verletzungen durch ein Bewegungsprogramm. Dieses Ergebnis ist durchaus erklärbar, denn Bewegung kann das Risiko zu stürzen und sich dabei zu verletzen erhöhen. Eine neuere Studie mit pflegebedürftigen Personen über 70 Jahre zeigt, dass die Überprüfung und Anpassung der Sehleistung (Brille, Augenoperation)sogar zu mehr Stürzen führen kann. Die häufigeren Stürze treten

Ebrahim, 1997

Latham, 2003

Cumming, 2007

eventuell durch ein verändertes Verhalten der Senioren auf: Die Senioren können wieder besser sehen, sind möglicherweise nicht mehr so vorsichtig wie vorher und stürzen deswegen öfter.

Zusammenfassung:

Wenn Sie sich entschieden haben, Sturz-vorbeugend aktiv zu werden:
Folgende Maßnahmen können **das Sturzrisiko** und teilweise das Risiko für **Sturz-bedingte Verletzungen** nachgewiesenermaßen senken:

- Übung von Kraft und Gleichgewicht bei Senioren mit erhöhtem Sturzrisiko; hierdurch wird auch das Risiko für Sturz-bedingte Verletzungen gesenkt.
- Bewegungsübungen, vor allem wenn sie häufig und über eine längere Zeit durchgeführt werden, das Gleichgewicht speziell trainieren und kein Walking-Programm (schnelles Gehen) beinhalten.
- Verbesserung der Sicherheit in der Wohnung insbesondere bei Senioren, die bereits gestürzt sind.
- Kombination aus Bewegungsübungen, Korrektur der Sehleistung und Verbesserung der Sicherheit in der Wohnung.
- Kombination aus Bewegungsübungen und Korrektur der Sehleistung.
- Untersuchung des Sturzrisikos mit anschließendem Angebot individuell angepasster Maßnahmen. Hierdurch wird auch das Risiko für Sturz-bedingte Verletzungen gesenkt.

Sturz-Vorbeugung scheint erfolgreicher zu sein, wenn ein erhöhtes Sturzrisiko vorliegt.

5. Zum Abschluss

Wir hoffen, dass diese Broschüre Sie über Risiko und Vermeidung von Stürzen und Sturz-bedingten Verletzungen informieren konnte.

Haben Sie Änderungsvorschläge oder Rückmeldungswünsche? Wir freuen uns über Ihre Anregungen!

Nachfolgend finden Sie noch Hinweise auf weiterführende Literatur und Adressen, den Nachweis der verwendeten Literatur in dieser Broschüre sowie eine Erklärung der verwendeten Fachbegriffe.

6. Weiterführende Literatur und Adressen

6.1 Informationen und Broschüren für Verbraucher

Geriatrisches Zentrum Ulm/Alb-Donau Bethesda:
Kraft- und Balance-Training – Programm des Geriatrischen Zentrums Ulm/Alb-Donau.
Geriatrische Klinik Ulm, Zollernring 26–28, 89073 Ulm,
Internet: www.aktivinjedemalter.de

Hrsg.: AOK Bundesverband:
Fit im Alter, 2005.
AOK Bundesverband, Rosenthaler Straße 31, 10178 Berlin,
Internet: www.aok-bv.de

Deutsche Gesellschaft für Allgemeinmedizin:
Leitlinie Ältere Sturzpatienten.
Internet: www.degam.de/leitlinien/4_sturz.html
Inklusive Patienteninformation

Becker C, Lindemann U, Rißmann U, Warnke A:
Sturzprophylaxe.
2. Auflage 2006. Hannover: Vincentz Verlag

Techniker Krankenkasse:
Osteoporose. Eine Information für Patienten und Angehörige. 2004.
Internet: www.akdae.de/45/Osteoporose.pdf

Schewe H:
Starke Füße, da steh ich drauf.
2007. Stuttgart: Trias Verlag

Internet-Adressen
Die Krankenkasse „BKK Vor Ort“ bietet auf ihrer Homepage eine Checkliste zur Sturz-Vorbeugung an. Die Checkliste listet Sturzrisiken in der Wohnung auf.
Internet: **https://www.bkkvorort.de/magazin/senioren/checkliste-sturzpraevention**

Der „Gesundfuchs“ vom Institut für Qualität und Wirtschaftlichkeit im Gesundheitswesen (IQWIG) erstellt Gesundheitsinformationen für Bürger und Patienten. Die Informationen beruhen auf aktuellen Forschungsarbeiten. Unter den folgenden Internet-Adressen werden Merkblätter zum Ausdrucken angeboten.
Internet: **http://www.gesundheitsinformation.de/vorbeugung-wie-kann-man-aeltere-menschen-vor-stuerzen-schuetzen.202.de.html**

http://www.gesundheitsinformation.de/merkblatt-vermeidung-von-stuerzen-bei-aelteren-menschen.203.de.html

6.2 Ansprechpartner

Je nach Region gibt es unterschiedliche Ansprechpartner.

Mögliche Ansprechpartner vor Ort:

- Städtische Einrichtungen wie Seniorenberatungsstellen in Sozial- und Wohnungsamt, Pflegebüros,
- Wohlfahrtsverbände (AWO, Caritas, Diakonie, DRK, Paritätischer Wohlfahrtsverband, Zentralwohlfahrtsstelle der Juden in Deutschland)
- Selbsthilfegruppen Osteoporose
- Krankenkassen
- Hausarzt
- Sportgruppen

7. Erklärung verwendeter Fachbegriffe

Knochendichtemessung

Bei der Knochendichtemessung wird geprüft, wie viel Knochenmasse der Knochen aufweist. Die Knochenmasse wird anhand des Kalksalzgehalts (Mineralsalzgehalts) des Knochens gemessen. Die Knochendichtemessung wird besonders bei Verdacht auf Osteoporose durchgeführt. Dabei werden abgeschwächte Röntgenstrahlen durch den Oberschenkel- und den Lendenwirbelknochen gesendet.

Oberschenkelhalsbruch

Der Oberschenkelknochen besteht aus einem langen Schaft, einem kurzen, abgewinkelten Hals und einem Hüftkopf. Aufgrund einer Entkalkung im Alter ist der Knochen im Halsbereich anfällig für Brüche.

Randomisiert-kontrollierte Studie

Der Nutzen von Sturz-vorbeugenden Maßnahmen muss in so genannten kontrollierten Studien geprüft werden. Diese Studien werden folgendermaßen durchgeführt: Einem Teil der Studienteilnehmer, der so genannten Behandlungsgruppe, werden vorbeugende Maßnahmen angeboten (zum Beispiel: Umgestaltung der Wohnumgebung, Bewegungsübungen). Dem

anderen Teil der Studienteilnehmer, der so genannten Vergleichsgruppe, werden keine Maßnahmen angeboten. Die Entscheidung, welcher Teilnehmer in welche Gruppe kommt, wird am besten per Zufall („randomisiert“) getroffen. Damit soll erreicht werden, dass die beiden Gruppen bei Studienbeginn vergleichbar sind. Nur so können Unterschiede in der Sturzhäufigkeit am Ende der Studie auf die angebotenen Maßnahmen zurück geführt werden. Solche Studien werden als „randomisiert-kontrollierte Studien“ bezeichnet. Neben der Nützlichkeit muss die Sicherheit der Maßnahme geprüft werden. Das heißt, es muss untersucht werden, ob Nebenwirkungen (unerwünschte Folgen) auftreten können.

Risikofaktor, Risikoindikator

Risikofaktoren sind Merkmale, die in einem statistisch nachgewiesenen Zusammenhang zu einer Erkrankung stehen. Ob dieser Zusammenhang tatsächlich ursächlich ist, das heißt ob das gefundene Merkmal die Erkrankung verursacht, ist dabei zunächst unklar. Ein Beispiel soll dies verdeutlichen: Viele Studien zeigen, dass Senioren, die bereits ein oder mehrmals gestürzt sind, häufiger stürzen als Senioren, die noch nicht gestürzt sind. Vorausgegangene Stürze sind jedoch keine „Ursache“

für folgende Stürze. Sie zeigen lediglich an, dass Merkmale oder Bedingungen vorliegen, die zu einer erhöhten Sturzwahrscheinlichkeit führen. Das Merkmal „bereits in der Vergangenheit gestürzt“ ist also ein Risikoindikator: Es zeigt ein erhöhtes Risiko an.

Tai Chi

Das Tai Chi kommt aus China, wo es ein Volkssport ist. Beim Tai Chi werden Bewegungsabfolgen eingeübt. Diese Art von Gymnastik entspannt die Muskulatur, die Gelenke werden beweglicher und die Körperhaltung wird verbessert. Ursprünglich war Tai Chi eine Kampfkunst zur Selbstverteidigung. Heute steht die allgemeine Bewegungslehre im Vordergrund.

Übersichtsarbeit

Eine systematische Übersichtsarbeit (Metaanalyse) fasst die Ergebnisse mehrerer randomisiert-kontrollierter Studien zusammen. Ziel ist es, die Aussagekraft von Studienergebnissen zu bündeln. Diese Zusammenführung ist nicht unproblematisch, da sich die einzelnen Studien zum Beispiel in der Studiendauer, in den angewendeten Maßnahmen oder in den Eigenschaften der Studienteilnehmer unterscheiden können. Dennoch gilt das Ergebnis

einer Übersichtsarbeit in der Regel als aussagekräftiger als das einer einzelnen Studie.

Unsicherheitsbereich

Da der Zufall es so will, würde in jeder Studie zur gleichen Fragestellung ein mehr oder weniger abweichendes Ergebnis erzielt werden. Man kann sich den 95% Unsicherheitsbereich so vorstellen: Hier würden 95 von 100 Ergebnisse einer Studie liegen, wenn diese Studie 100 mal unter den gleichen Bedingungen durchgeführt würde.

Wahrscheinlichkeit

Ein Maß für die Wahrscheinlichkeit ist die anteilige Häufigkeit eines Ereignisses, bezogen auf die Gesamtgruppe. Zum Beispiel: Der Anteil der Senioren, die in einem bestimmten Zeitraum einen Sturz erleiden, bezogen auf alle Senioren: pro Jahr stürzen 30 von 100 Senioren: Die Wahrscheinlichkeit innerhalb eines Jahres zu stürzen beträgt 30%.

Wahrscheinlichkeitsverhältnis

Der Begriff Wahrscheinlichkeitsverhältnis soll an einem Beispiel aus der *Tabelle 3* veranschaulicht werden. Als Risikofaktor für einen Sturz wird in der

Tabelle ein „Sturz im letzten Jahr“ mit dem Wahrscheinlichkeitsverhältnis von 2,8 angegeben. Ein Wahrscheinlichkeitsverhältnis von 2,8 bedeutet, dass ein Sturz 2,8-mal wahrscheinlicher Senioren betrifft, die im letzten Jahr gestürzt sind, als Senioren, die nicht gestürzt sind.

8. Verwendete Literatur

BAuA, Dortmund, Repräsentative Haushaltsbefragung für Deutschland in den Jahren 2000/2001

Campbell AJ, Robertson MC: Rethinking individual and community fall prevention strategies: a meta-regression comparing single and multifactorial intervention. Age Ageing 2007; 36: 656–662

Cumming RG, Ivers R, Clemson L, Gullen J, Hayes MF, BOptom MT, Mitchell P: Improving Vision to Prevent Falls in Frail Older People: A Randomized Trial. JAGS 2007; 2: 175–181

Ebrahim S, Thompson PW, Baskaran, Evans K: Randomised placebo-controlled trial of brisk walking in the prevention of postmenopausal osteoporosis. Age Ageing 1997; 26: 253–260

Elley CR, Robertson MC, Garrett S, Kerse NM, McKinllay E, Lawton B, Moriarty H, Moyes SA, Campbell AJ: Effectiveness of a Falls-and-Fracture Nurse Coordinator to Reduce Falls: A Randomized, controlled Trial of At-Risk Older Adults. JAGS 2008; 8: 1383–1389

Ganz DA, Bao Y, Shekelle PG, Rubenstein LZ: Will my patient fall? JAMA 2007; 297: 77–86

Gillespie LD, Gillespie WJ, Robertson MC, Lamb SE, Cumming RG, Rowe BH: Interventions for preventing falls in elderly people (Cochrane Review). The Cochrane Library, Issue 2, 2003. Oxford: Update Software Ltd

Harvard School of Public Health. http://www.hsph.harvard.edu/healthliteracy/overview.html

Hendriks MRC, Bleijlevens MHC, van Haastregt JCM, Crebolder HFJM, Diederiks JPM, Evers SMAA, Mulder WJ, Kempen GIJM, van Rossum E, Ruijgrok JM, Stalenhoef PA, van Eijk, JTM: Lack of Effectiveness of a Multidisciplinary Fall-Prevention Program in Elderly People at Risk: A Randomized, Controlled Trial. JAGS 2008; 8: 1390–1397

Icks A, Haastert B, Wildner M, Becker C, Meyer G: Trend of hip fracture incidence in Germany 1995-2004: a population-based study. Osteoporos Int 2008; 8: 1139–1145

Icks A, Haastert B, Wildner M, Becker C, Meyer G: Inzidenz von Hüftfrakturen in Deutschland - Auswertung der Krankenhausdiagnosestatisik 2004. Dtsch Med Wochenschr 2008; 133:125–128

Latham NK, Anderson CS, Lee A, Bennett DA, Moseley A, Cameron ID: A randomized, controlled trial of quadriceps resistance exercise and vitamin D in frail older people: the Frailty Interventions Trial in Elderly Subjects (FITNESS). JAGS 2003; 51: 291–299

Meyer G, Köpke S: Interventionen zur Prävention von Stürzen und sturzbedingten Verletzungen: die beste Evidenz für eine komplexe Problemlösung. In: Behrens J, Langer G (Hrsg.) Externe Evidence und klinische Pfade. Handbuch Evidence-based Nursing. Bern: Hans Huber (im Druck 2009)

Nguyen ND, Frost SA, Center JR, Eisman JA, Nguyen TV: Development of a nomogram for individualizing hip fracture risk in men and women. Osteoporos Int 2007; 18: 1109–1117

Parker MJ, Gillespie WJ, Gillespie LD: Effectiveness of hip protectors for preventing hip fractures in elderly people: systematic review. BMJ 2006; 332: 571–574

Pientka L, Friedrich C: Die Kosten hüftgelenksnaher Frakturen in Deutschland: Eine prospektive Untersuchung. Z Gerontol Geriat 1999; 32: 326–332

Sherrington C, Whitney JC, Lord SR, Herbert RD, Cumming RG, Close JCT: JAGS 2008; 12: 2234–2243

Steckelberg A, Berger B, Köpke S, Heesen C, Mühlhauser I: Kriterien für evidenzbasierte Patienteninformationen. Z Arztl Fortbild Qualitatssich 2005; 99: 343–351

Talbot LA, Musiol RJ, Witham EK, Metter EJ: Falls in young, middle-aged and older community dwelling adults: perceived cause, environmental factors and injury. BMC Public Health 2005; 5: 86

Woolf AD, Akesson K: Preventing fractures in elderly people. BMJ 2003; 327: 89–95
